# Neighborhood-informed positional information for precise cell identity specification

**DOI:** 10.1038/s44320-026-00211-y

**Published:** 2026-05-05

**Authors:** Michal Erez, Roy Friedman, Mor Nitzan

**Affiliations:** 1 School of Computer Science and Engineering, The Hebrew University, Jerusalem, Israel; 2 Racah Institute of Physics, The Hebrew University, Jerusalem, Israel; 3 Faculty of Medicine, The Hebrew University, Jerusalem, Israel

**Keywords:** Chromatin, Transcription & Genomics, Computational Biology, Development

## Abstract

During development, cells reliably establish their identities, a process that is enabled in part by positional information encoded in gene expression patterns. Previous works showed that cells in *Drosophila* embryos can utilize this information to decode their position along the anterior-posterior axis with a 1% embryo-length positional precision. However, this precision is insufficient to uniquely determine position, leading to a positional information gap. Here, we propose a neighborhood-informed information-theoretic framework which allows to quantitatively estimate the amount of information regarding position which exists in the microenvironment of each cell. We formulate how much additional information exists in neighboring cells as a function of spatial variation in gene expression. We show that the additional information encoded by local neighborhoods is sufficient to uniquely specify cell identities, closing the information gap on average across major patterning axes in *Drosophila* embryos, gastruloids, and the developing neural tube. Furthermore, neighborhood-informed decoders predict cell positions and downstream gene expression patterns more accurately than cell-independent decoders, resulting in lower decoding variability, which is maintained in mutant *Drosophila* embryos. Our results provide a basis for the analysis of cellular decision-making in the context of their microenvironments.

## Synopsis

The behavior and fate of cells in the developing embryo depends on their position. Incorporating information from local microenvironments boosts the amount of positional information available to cells, enough to uniquely specify location along patterning axes of multiple biological systems.

- The positional information contained in local microenvironments allows unique position specification along the patterning axes of Drosophila embryos, gastruloids and the developing neural tube.
- Decoding position when informed by neighbors significantly reduces uncertainty of position relative to cell-independent decoding.
- Utilizing information about morphogen expression levels in neighbors allows for better decoding of cellular identities.

## Abstract (web summary)

The behavior and fate of cells in the developing embryo depends on their position. Incorporating information from local microenvironments boosts the amount of positional information available to cells, enough to uniquely specify location along patterning axes of multiple biological systems.

## Graphical abstract

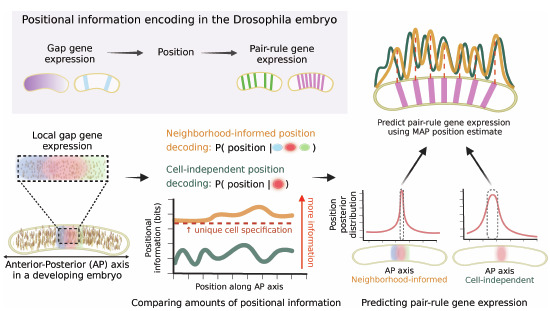

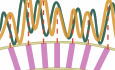

## Introduction

Multicellular self-organization during development is an incredibly precise and reproducible process (Arias and Hayward, 2006; Bentovim et al, 2017; Gregor et al, 2007; Little et al, 2013; Petkova et al, 2014). In several developmental contexts, the precision of self-organization is derived from positional information, where extrinsic signals, such as morphogens, carry information that enables cells to effectively decipher their position within an embryo or within a tissue. This positional information, in turn, can be used by cells to specify their identity as a function of their position (Dubuis et al, 2013b; Houchmandzadeh et al, 2002; McGough et al, 2024; Petkova et al, 2019; Wolpert, 1969, 1971).

In recent years, a seminal line of works formalized an information-theoretic framework for positional information, as well as its accuracy and limitations, with a focus on positional information of individual cells in the well-studied model of the *Drosophila* embryo (Dubuis et al, 2013b; McGough et al, 2024; Tkačik et al, 2015; Tkačik and Gregor, 2021). The developmental dynamics in *Drosophila* embryos, where gene expression patterns and the different stages of development have largely been mapped out (Arbeitman et al, 2002; Gaul and Jäckle, 1990; Jäckle et al, 1986), provide a useful test case for the study of positional information (Dubuis et al, 2013b; Jaeger et al, 2004; McGough et al, 2024; Petkova et al, 2019; Tkačik et al, 2015; Tkačik and Gregor, 2021). In these works, positional information was studied along the main body axis of the embryo, the anterior–posterior (AP) axis, where information from maternal morphogens provides input to an interacting network of *gap genes* {knirps (kni), Krüppel (Kr), hunchback (Hb), giant (Gt)}, whose expression in turn provides input to the patterned striped expression of *pair-rule genes* {Eve (eve), paired (prd), runt (run)}. The pair-rule genes, thereby, map the segmented body plan of the developed fly. Given this flow of information, it was suggested that expression patterns of gap genes encode positional information, and expression of pair-rule genes can be considered as the positional readout of the cells (Carroll, 1990; Rivera-Pomar and Jackle, 1996). This focus on *Drosophila* embryos is guided by high reproducibility in the expression of genes during development across numerous individuals (Lawrence, 1992). The level of reproducibility is such that the variance of stripe position between different individuals is approximately half the internuclear distance (Dubuis et al, 2013a). The choice for studying the one-dimensional AP axis on its own is guided by previous observations that patterning signals along the dorsal–ventral (DV) and AP axes are largely independent, and that patterning along the AP axis underlies later developmental patterning and structural formation of the *Drosophila* embryo (Nüsslein-Volhard, 1991). Further, the collection of high-resolution immunofluorescence microscopy data, jointly staining multiple proteins, in specific developmental periods for a large number of replicates, has proved invaluable for an information-theoretic view of positional information (Dubuis et al, 2013b).

Indeed, gap gene expression provides individual cells in the *Drosophila* embryo enough information to decode their position along the AP axis with high precision (Petkova et al, 2019). However, this *cell-independent* decoding is insufficient to fully specify unique cell positions (McGough et al, 2024). In other words, there seems to be an *information gap* between the information available to individual cells and the information required for unique cell localization (McGough et al, 2024). This does not align with empirical observations, which suggest that sharp striping patterns of pair-rule genes in *Drosophila* embryos require localization at single-nuclei resolution across the AP axis (McGough et al, 2024). It was suggested that the information gap can be theoretically bridged by long-range gene expression correlations along the AP axis (McGough et al, 2024). This is because such gene expression correlations introduce effective constraints: they reduce the number of possible collective (AP axis-wide) gene expression states, and thus, reduce the amount of information needed for each cell to specify its position. It remains unclear, however, if this information is locally available to cells.

Here, we suggest a formulation for positional information encoding that explicitly considers information regarding position existing in the local cellular microenvironment of the cell via a *neighborhood-informed* positional information encoding framework. We show that such neighborhood-informed positional decoding can close the positional information gap, namely, that sufficient information exists in their local neighborhoods to uniquely decode the positions of cells along the AP axis.

First, we show that the long-range correlations mentioned above can be viewed as a consequence of correlations in gene expression between neighboring cells. These local expression correlations completely dictate long-range correlations discussed in previous work (McGough et al, 2024), and above a certain threshold, can close the information gap. Empirically, we find that these pairwise correlations are indeed higher than the theoretical bound, based on measurements of gene expression along the AP axis in *Drosophila* embryos (Petkova et al, 2019). This result indicates that the information missing for cells to uniquely specify their position exists in their local neighborhood, but does not allow for an explainable quantification of the amount of information locally available. We address this via a *neighborhood-informed* positional information framework. In our framework, the amount of positional information encoded in local expression levels can be inferred from the performance of the optimal probabilistic position decoder given the gene expression in a certain position and its microenvironment. Formally, the neighborhood-informed probabilistic position decoder is defined as $P(\hat{x}| g,{g}_{n})$ where $\hat{x}$ is the predicted position given the gene expression at a specific position, *g*, and that of its neighborhood, *g*_*n*_. We view the gene expression profile of a cell as a description of its *state* (Tkačik and Gregor, 2021), which can be potentially transmitted between cells either directly or indirectly. The neighborhood-informed positional decoder $P(\hat{x}| g,{g}_{n})$ is contrasted to a cell-independent positional decoder $P(\hat{x}| g)$, which only has access to the cell’s own transcriptomic state *g*. In both cases, the amount of information that can be extracted regarding position is inversely proportional to the variance in the positional prediction (Dubuis et al, 2013b). Through this framework, we are able to mathematically formulate how much additional positional information can be gained by incorporating information from neighbors. We find that the variance of the positional prediction using a neighborhood-informed decoder is consistently low enough to close the gap in positional information, unlike the cell-independent decoder (Petkova et al, 2019). Furthermore, we demonstrate that the neighborhood-informed decoded positions can better predict pair-rule gene expression profiles.

We then test our framework in the context of mutant *Drosophila* embryos, where the maternal input has been perturbed (Petkova et al, 2019). Pair-rule gene expression in such mutants, similarly to wild-type embryos, is approximately determined by gap gene expression profiles (Petkova et al, 2019). We re-evaluate the position decoding in these maternal input perturbed embryos to include neighborhood information, assuming an encoder that was optimized in wild-type embryos. In this case as well, the neighborhood-informed decoder maps gap gene expression to pair-rule gene expression with higher positional certainty than a cell-independent decoder. These results provide additional support that cells may utilize neighborhood information directly to infer cell state, such as position, and provide a framework to test neighborhood-informed positional information encoding in diverse biological contexts.

Finally, we explore our formulation of the neighborhood-informed decoder beyond the biology of *Drosophila* embryos. To do so, we consider gastruloids, an in vitro model for early mammalian development (Merle et al, 2024), and the developing neural tube in mice (Zagorski et al, 2017), both of which were previously analyzed within the framework of positional information (Tkačik et al, 2015) along a single axis of development. When analyzing the positional information along the AP axis of gastruloids (Merle et al, 2024) and the dorso-ventral (DV) axis of the developing neural tube (Zagorski et al, 2017), we again find that the neighborhood-informed decoder is able to specify unique cell locations along most of the patterning axis, as opposed to the cell-independent decoder, showcasing the generality of our framework to different developmental contexts.

## Results

### Short-range gene expression correlations close the positional information gap in the *Drosophila* embryo

One way to ascertain the amount of information pertaining to position that exists within gene expressions is to consider a decoder that predicts the location of the cell $\hat{x}$, given the expression levels of the morphogens, *g*. The amount of positional information in the gene expression is then a function of the entropy of the decoder’s distribution over predicted locations when there is access to the gene expression, $P(\hat{x}| g)$, versus without, $P(\hat{x})$ (Dubuis et al, 2013b). By further assuming that the position decoder is optimal, i.e., has the lowest possible amount of uncertainty regarding the cell position when using the signals available to each cell, this analysis allows us to ask whether it is plausible that cells predict their position in a developmental context (Petkova et al, 2019). Concretely, using an information-theoretic view, we can ask: Is enough information regarding location encoded in the expression of a set of genes to enable the specification of cell fate by predicting the location?

Accordingly, to estimate the amount of positional information encoded in gene expression patterns, we analyze a position predictor whose only input is the gene expression levels. As such, the decoded position $\hat{x}$ depends on observed gene expression levels, *g*, at the (true) position *x*. Under the assumption that $\hat{x}=x+\eta$ where *η* is Gaussian distributed, then the amount of information that the expression levels of gap genes contain regarding the true position can be written in closed form and is inversely related to the error of the position estimator, namely the variance ${\sigma }^{2}(\hat{x}| g)$ of the predicted location. The amount of positional information *I*_position_ that can be extracted from gene expression levels along the length of an embryo can be formulated as (Dubuis et al, 2013b):

1 $${I}_{{{{\rm{position}}}}}={\log }_{2}L-{\log }_{2}\left(\sqrt{2\pi e}\cdot \sigma (\hat{x}| g)\right)$$

where *L* is the length of the embryo. It was previously shown in *Drosophila* embryos that there exists enough information in the local expression levels of gap genes along the AP axis to estimate the position of a cell with an accuracy of ~1% of the embryo’s length (Dubuis et al, 2013b; Tkačik et al, 2015). While 1% precision seems extremely accurate, it is insufficient for the task of uniquely determining cell position in *Drosophila* embryos (McGough et al, 2024). This lack of information is encapsulated in the variance of the position estimate and is formalized through the *information gap* (McGough et al, 2024):

2 $${I}_{{{{\rm{gap}}}}}={I}_{{{{\rm{unique}}}}}-{I}_{{{{\rm{position}}}}}={\log }_{2}\left(\frac{N}{L}\sqrt{2\pi e}\cdot \sigma (\hat{x}| g)\right)$$

where ${I}_{{{{\rm{unique}}}}}={\log }_{2}N$ is the amount of information required to uniquely identify cellular position, and *N* is the number of nuclei along the AP axis. As long as *I*_gap_ is greater than 0, it is possible, for example, for the predicted ordering of two adjacent cells to flip.

The functional forms of the positional information and information gap above do not incorporate any assumptions on the structure of gap gene expression along the AP axis. In practice, however, these gene expression patterns vary smoothly as a function of position, and there exist long-range expression correlations along the AP axis (McGough et al, 2024). This correlation structure implies that genes in adjacent positions cannot have arbitrarily different expression levels relative to each other, effectively reducing the space of possible spatial expression patterns and predictions made using them. This constrained space of possible states means that less information is required per cell to determine its position.

To be more precise, McGough et al (2024) showed that the errors in the predicted position by each cell are correlated across the AP axis, in part due to correlations in the expression of the gap genes. This correlation structure was modeled as:

3 $${{{\rm{corr}}}}\left[{\hat{x}}_{n},{\hat{x}}_{n+\Delta x}\right]={e}^{-\frac{L}{\xi N}\Delta x}$$

where Δ*x* is the distance between two nuclei along the AP axis and ${\hat{x}}_{n}$ is the predicted position for the *n*th nucleus. The information gap is closed when $\xi \ge 19.5\frac{L}{N}$ (McGough et al, 2024). Intuitively, one way that such long-range correlations might arise is if each small, local environment along the AP axis is highly correlated. Indeed, we show that it is enough for pairs of cells to be highly correlated to induce long-range correlations, and Eq. (3) is recapitulated when (see Appendix):

4 $${{{\rm{corr}}}}\left[{\hat{x}}_{n},{\hat{x}}_{n+1}\right]={e}^{-\frac{L}{\xi N}}$$

As mentioned above, the positional information gap is closed when the length-scale *ξ* is large enough. Under Eq. (4), this translates to high correlations between the positional predictions for pairs of adjacent nuclei. In particular, correlations of ${{{\rm{corr}}}}\left[{\hat{x}}_{n},{\hat{x}}_{n+1}\right] \; > \; 0.95$ close the information gap. We will additionally note that the value of pairwise correlation must also be lower than 1, since otherwise there would be no additional positional information in the neighborhood due to its invariance along the AP axis.

**Fig. 1:**
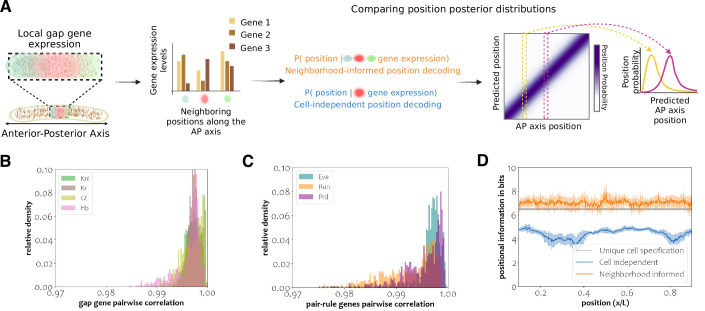
Positional information encoded in neighboring positions along the AP axis in *Drosophila* embryos. (**A**) A schematic diagram showing the general framework. Using gap gene expression at adjacent positions along the AP axis, we decode position in a neighborhood-informed manner and compare to cell-independent decoding. Decoding position results in positional distributions that can be represented as decoding maps (right). (**B**) The distribution of pairwise correlation in gap gene expression of adjacent positions for each of the four gap genes, Kni, Kr, Gt, and Hb. (**C**) The distribution of pairwise correlation in the expression of adjacent positions for each of the three pair-rule genes: Eve, Prd, and Run. (**D**) The positional information in bits along the AP axis using a neighborhood-informed decoder (orange) as opposed to a cell-independent decoder (blue). The gray dashed line represents the information needed for unique cell identification. Source data are available online for this figure.

To empirically verify whether such correlations exist in wild-type (WT) *Drosophila* embryos, we analyze embryos that were fluorescently stained against the four trunk gap genes and three pair-rule genes, collected by Petkova et al (2019) (see “Methods” for more information regarding data). In *Drosophila* embryos, gap genes are thought of as encoders of positional information, while the pair-rule genes are often considered the positional readouts (Petkova et al, 2019). As there is no direct access to the true prediction of position for each nucleus, we analyze pairwise correlations for the expression levels of both gap genes and pair-rule genes as proxies. When analyzing these gene expression profiles, we find that the pairwise correlations in expression of both gap and pair-rule genes are indeed above 0.95 across the full AP axis (Fig. 1B,C).

As stated above, McGough et al (2024) showed that long-range correlations in the decoding of cell locations along the AP axis can lead to the unique specification of position. However, we do not expect that one cell along the AP axis has access to information regarding the predicted location of another cell 19.5% down the AP axis. Our analysis above shows that it is enough if such correlations exist at the distance of single neighbors, a distance across which cells are expected to be able to interact, either directly or indirectly. This motivates a shift in perspective, from considering the information available to each cell independently, to studying what information exists in small local environments comprised of a cell and its neighbors.

### Neighborhood information reduces positional error in the *Drosophila* embryo

In the previous section, we have established that at the embryo level the combination of positional information in each cell along with the positional information encoded in short-range, pairwise gene expression correlations allows for unique cell localization. Effectively, such short-range expression correlations constrain the potential space of spatial expression patterns, which as a result, reduces the extent of positional information required by each cell to uniquely specify its position. In the following, we formulate the encoding of positional information by explicitly incorporating information about neighboring gene expression, and analyze the resulting variance in the predicted position ${\sigma }^{2}(\hat{x}| g,{g}_{n})$ (see “Methods”), where *g*_*n*_ represents gene expression levels in a cell’s neighborhood.

Specifically, the neighborhood-informed positional error can be characterized (analogously to the cell-independent positional error (Dubuis et al, 2013b)) by:

5 $$\frac{1}{{\sigma }^{2}(\hat{x}| {g}_{{{{\rm{env}}}}})}={\Sigma }_{i,j=1}^{d}{\left[\frac{d{\overline{g}}_{{{{\rm{env}}}}}^{(i)}(x)}{dx}{({C}_{{{{\rm{env}}}}}{(x)}^{-1})}_{ij}\frac{d{\overline{g}}_{{{{\rm{env}}}}}^{(j)}(x)}{dx}\right]}_{x={\max} _{\hat{x}}P(\hat{x}| {g}_{{{{\rm{env}}}}})}$$

where *g*_env_ = (*g*, *g*_*n*_), *d* is the number of genes whose expressions are considered in *g*_env_, ${g}_{{{{\rm{env}}}}}^{(i)}$ is the expression level of *i*th gene, $\frac{d{\overline{g}}_{{{{\rm{env}}}}}^{(i)}(x)}{dx}$ is the mean derivative of the expression level as a function of *x*, and *C*_env_(*x*) is the covariance matrix of the gene expressions in *g*_env_. In general, additional information incorporated from neighboring positions is not expected to increase the error. In fact, we show that the positional error strictly decreases when adding neighboring information (Appendix):

6 $${\sigma }^{2}(\hat{x}| g,{g}_{n}) \; < \; {\sigma }^{2}(\hat{x}| g)$$

In other words, the neighborhood-informed variance of estimated position (left term in Eq. (6)) is bounded from above by the cell-independent variance of estimated position (right term). When the expression of gap genes follows a Gaussian distribution, which Dubuis et al (2013b) have shown to approximately hold, the difference between ${\sigma }^{2}(\hat{x}| g)$ and ${\sigma }^{2}(\hat{x}| g,{g}_{n})$ can be written in closed form (see Appendix).

To empirically demonstrate that further positional information exists in local neighborhoods, we analyze the neighborhood-informed decoder in wild-type *Drosophila* embryos, given the gap gene expressions against which they were fluoroscently stained (“Methods”). For the *n* = 38 WT embryos dataset, we find that the positional information in the neighborhood-informed decoder is sufficiently high to uniquely specify cell locations and close the positional information gap, unlike the cell-independent decoder (Fig. 1D). In particular, we show that the variance of the neighborhood-informed decoder is $\sigma (\hat{x}| g,{g}_{n}) \sim 0.00195L$, almost an order of magnitude smaller than the variance of the cell-independent decoder, $\sigma (\hat{x}| g) \sim 0.01L$. These variances correspond to an average of *I*_NI_ = 7.0 ± 0.5 bits/cell of positional information when using the neighborhood-informed decoder versus *I*_CI_ = 4.4 ± 0.5 bits/cell for the cell-independent decoder (“Methods”). As such, the amount of positional information accessible by the neighborhood-informed decoder is larger than that needed to uniquely specify location, *I*_unique_ = 6.49 ± 0.06 bits/cell (McGough et al, 2024), whereas the cell-independent decoder does not close the information gap.

Altogether, while these results are limited by the set of genes empirically measured, they demonstrate that a cell’s local microenvironment contains positional information which is unavailable to each cell independently, and enables the unique specification of cellular location. Therefore, in contrast to cell-independent positional encoding, our results show that neighborhood-informed positional decoding is sufficient to uniquely position a cell along the AP axis.

### Neighborhood-informed position decoding in wild-type Drosophila embryos

Beyond variances, we can analyze the position decoder directly, which will then enable prediction of downstream events, such as pair-rule gene expression patterns. Using Bayes’ law, the full distribution $P(\hat{x}| {g}_{{{{\rm{env}}}}})$ over the predicted position $\hat{x}$ given gap gene expression ${g}_{{{{\rm{env}}}}}\equiv {[g,{g}_{n}]}^{T}$ at position *x* and its neighboring environment is (Fig. 1A, “Methods”):

7 $$P(\hat{x}| {g}_{{{{\rm{env}}}}})=\frac{1}{Z({g}_{{{{\rm{env}}}}})}P({g}_{{{{\rm{env}}}}}| \hat{x}){P}_{x}(\hat{x})$$

where ${P}_{x}(\hat{x})$ is the prior distribution over positions, $P({g}_{{{{\rm{env}}}}}| \hat{x})$ is the likelihood of *g*_env_ at position $\hat{x}$, and $\frac{1}{Z({g}_{{{{\rm{env}}}}})}$ is a normalizing factor.

**Fig. 2:**
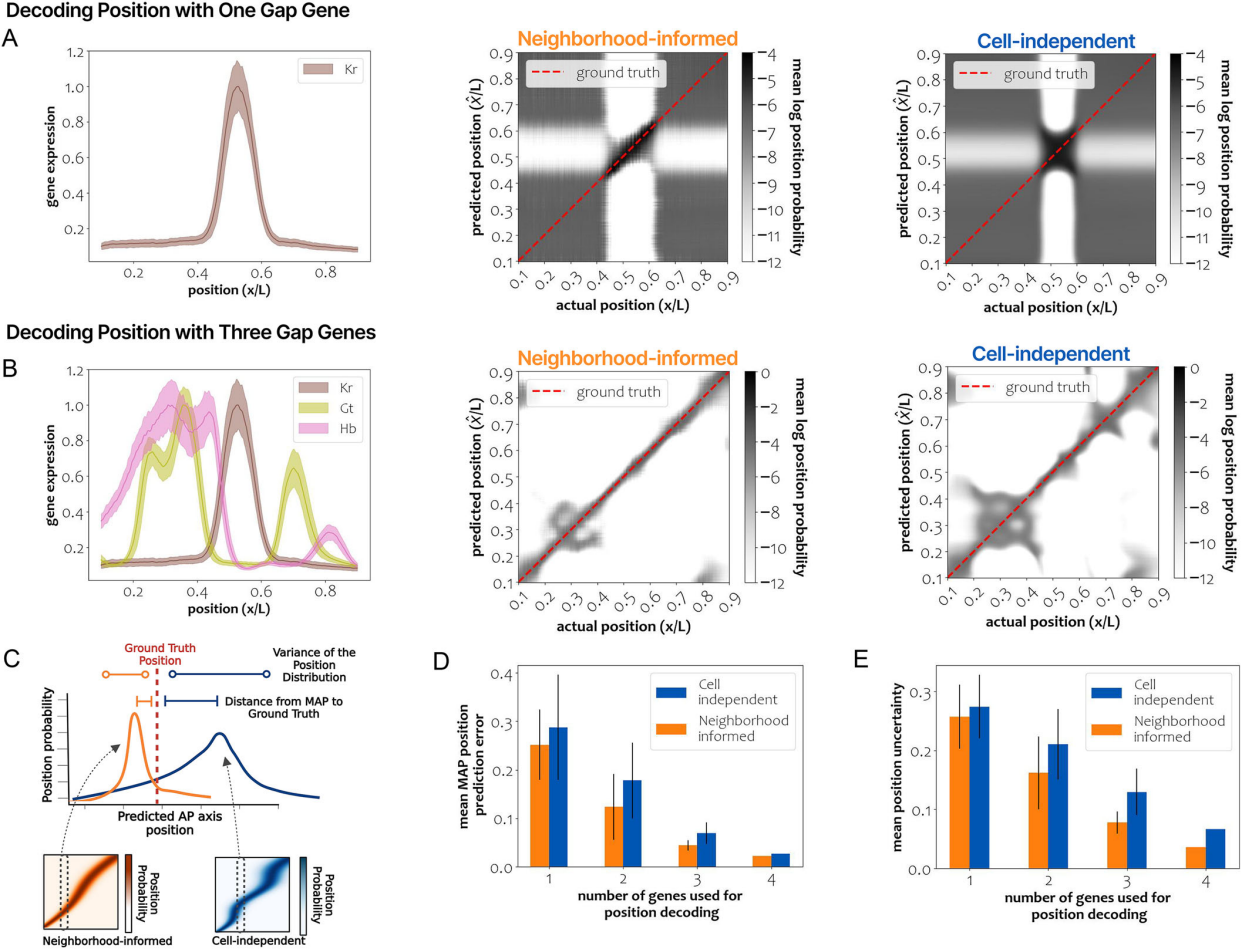
Decoding position from gap gene expression in *Drosophila* embryos. (**A**) (left) gap gene expression of Kr along the AP axis; neighborhood-informed (middle) and cell-independent (right) position decoding maps based on Kr gene expression. (**B**) (left) gap gene expression of Kr, Gt, Hb along the AP axis; neighborhood-informed (middle) and cell-independent (right) position decoding maps based on the expression of these three genes. (**C**) Schematic diagram portraying the comparison between the neighborhood-informed versus cell-independent position decoding distributions. (**D**) The mean error of position prediction using the MAP estimate over *n*_WT_ = 38 embryos, grouped by the number of gap genes used for decoding. Error bars represent a single standard deviation over the choice of genes used for position decoding. For each group, we compare neighborhood-informed to cell-independent decoding. The predicted error is significantly lower for neighborhood-informed decoding (*t* test *P* values of 0.01, 10^−10^, 3 × 10^−12^ and 2 × 10^−6^ when comparing MAP prediction errors across the AP axis for [1, 2, 3, 4] genes, respectively, and statistically significant in all embryo-to-embryo comparisons with 2, 3, and 4 genes). Decoding with one gene results in a statistically significant improvement in 75% of embryos. (**E**) The average standard deviation of the predicted position distribution over *n*_WT_ = 38 embryos, grouped by the number of gap genes used for decoding position, is significantly lower for neighborhood-informed decoding (*t* test *P* values of 4 ⋅ 10^−5^, 3 × 10^−7^ and 4 × 10^−9^ when comparing between the standard deviation along the AP axis when using [2, 3, 4] genes respectively, and are statistically significant in all individual 38 embryo-to-embryo comparisons for all 15 subsets of genes). Error bars represent a single standard deviation over the choice of genes used for position decoding. Source data are available online for this figure.

Based on this, we can compute the posterior distribution of predicted positions, i.e., the *positional distribution*, given the gene expression at every position along the AP axis. When training the decoder on 254 WT embryos collected in (Petkova et al, 2019) and predicting the posterior distribution for a separate dataset composed of 38 WT embryos collected in the same work, the neighborhood-informed positional distribution is more strongly peaked around the ground-truth positions relative to the cell-independent positional distribution (Fig. 2A,B), showcasing the increased accuracy in maximum-a-posteriori (MAP) prediction as well as the reduction in the ambiguity over predicted positions when utilizing neighborhood information (Fig. 2C–E). The results are consistent when both training and decoding of positions are performed on the same dataset (Appendix Fig. S4).

While the positional decoding prediction error and the standard deviation of the positional distribution both decrease with increasing number of gap genes used for decoding for both decoder types (neighborhood-informed and cell-independent), the neighborhood-informed decoder consistently outperforms the independent decoder (Fig. 2D,E). In particular, we find that for all choices of gene subsets, this difference is statistically significant when comparing the distribution of errors across embryos.

### Prediction of pair-rule gene expression

**Fig. 3:**
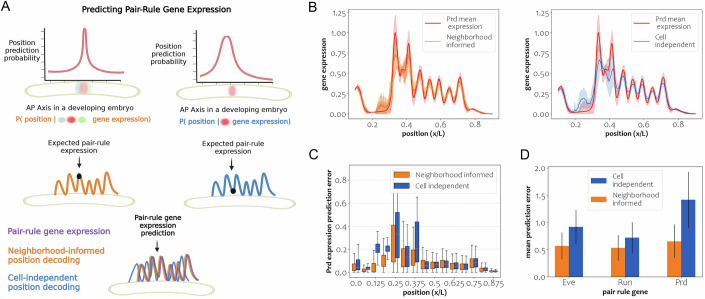
Pair-rule stripe reconstruction in WT *Drosophila* embryos. (**A**) A schematic diagram of pair-rule gene expression prediction based on positional information. (**B**) Reconstruction of the pair-rule gene Prd based on a neighborhood-informed (left) and a cell-independent (right) decoder. (**C**) Prd reconstruction error binned over 20 positions across the AP axis, over *n*_PR_ = 34 WT embryos. Boxplots are defined between the 25% and 75% percentiles, with the center line depicting the median and whiskers  × 1.5 the interquartile range below and above the box. (**D**) The mean reconstruction error per pair-rule gene, averaged over embryos and positions, divided by the standard deviation per position, is significantly lower for neighborhood-informed decoding (*t* test *P* values when comparing the average prediction error for cell-independent and neighborhood-informed decoders: Eve = 8 × 10^−7^, Run = 2 × 10^−3^ and Prd = 3 × 10^−11^). Source data are available online for this figure.

Next, we will directly characterize the transition from gap gene expression to pair-rule gene expression, considered as the positional readout of the cell (Fig. 3A). In other words, do gap gene expression profiles in local microenvironments encode the information necessary to specify pair-rule gene expression patterns?

We reconstruct pair-rule gene expression profiles by utilizing the posterior distribution over predicted positions given gap gene expression. The expected pair-rule gene expression $\widehat{{g}_{k}}$ at a certain position given gap gene expression in that position and its neighborhood, *g*_env_, is given by:

8 $${\mathbb{E}}[\widehat{{g}_{k}}| {g}_{{{{\rm{env}}}}}]={\Sigma }_{\hat{x}}P(\hat{x}| {g}_{{{{\rm{env}}}}}){ {\bar{g}} }_{k}(\hat{x}),$$

where ${\overline{g}}_{k}(\hat{x})$ is the mean over wild-type embryos’ expression of pair-rule gene *k* at position $\hat{x}$. An analogous expression can be obtained for the cell-independent decoder by replacing *g*_env_ with *g*.

In Fig. 3B, we show a qualitative comparison of the prediction of the expression of pair-rule gene Prd along the AP axis based on either the neighborhood-informed or cell-independent decoders, in comparison to its mean ground-truth expression (similar results for the additional pair-rule genes, Eve and Run, are shown in Appendix Fig. S1). The neighborhood-informed prediction is more accurate, relative to the cell-independent prediction, across the AP axis (Fig. 3C). This initial result generalizes, as the prediction error of pair-rule expression, averaged across all positions and wild-type embryos, is statistically significantly lower for the neighborhood-informed decoder, for all three pair-rule genes (Fig. 3D).

### Decoding position and predicting pair-rule stripes in mutant embryos

The empirical results from the previous sections show that substantial positional information is encoded in the local neighborhood and that an optimal decoder can decode position with higher accuracy and lower uncertainty when integrating neighborhood information. However, do cells necessarily decode position utilizing neighborhood information?

**Fig. 4:**
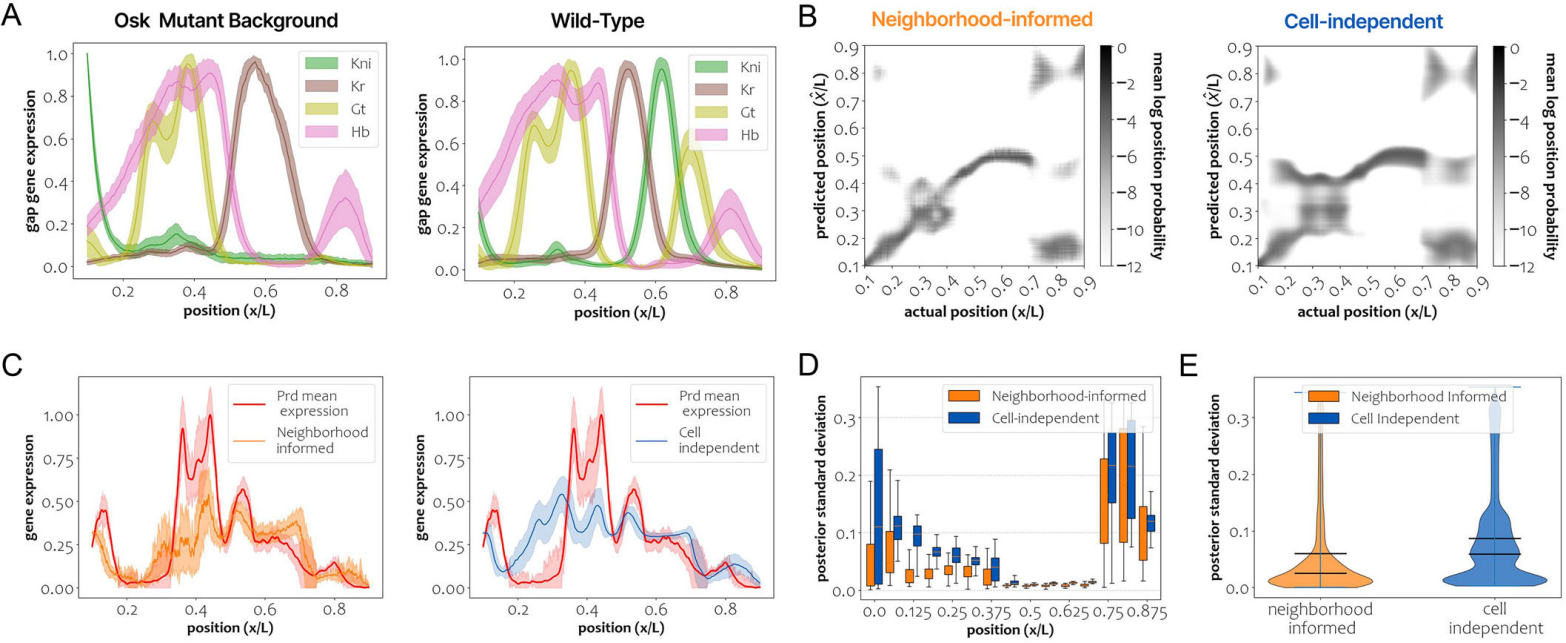
Neighborhood-informed position decoding and pair-rule stripe reconstruction in *osk* mutants. (**A**) Gap gene expression of all four gap genes in *osk* mutants (left) and in wild-type embryos (right). (**B**) Decoding maps given neighborhood-informed (left) and cell-independent (right) decoders. (**C**) Pair-rule expression prediction across the AP axis, using neighborhood-informed (left) and cell-independent (right) decoders. (**D**) Posterior standard deviation over predicted positions across 20 binned positions along the AP axis, calculated over *n*_*osk*_ = 28 embryos. Boxplots are defined between the 25 and 75% percentiles, with the center line depicting the median and whiskers  × 1.5 the interquartile range below and above the box. (**E**) Distributions over posterior standard deviation of predicted positions (over all embryos and all positions). Horizontal lines represent the 25%, 50%, and 75% quantiles. The standard deviation of the posterior for the neighborhood-informed decoder is lower than that of the cell-independent decoder, and the difference between the two distributions is statistically significant with a *P* value of 10^−86^ under a standard *t* test. Source data are available online for this figure.

One of the strongest tools to approach such a question is by testing the given hypotheses on a perturbed system. This is possible in *Drosophila* embryos through the use of perturbed maternal signals (Petkova et al, 2019), which alter the expression levels of both gap genes and pair-rule genes (“Methods”). Our underlying assumption, supported by previous work (Petkova et al, 2019), is that although such perturbations, or mutant backgrounds, affect the spatial expression patterns of gap genes (Fig. 4A), they do not substantially affect the wild-type positional decoder, and thus do not affect the mapping from gap gene expression to pair-rule gene expression, through the decoding of position (even though such decoded positions no longer reflect the actual position along the AP axis).

Neighborhood-informed position decoding in mutant embryos results in notably less ambiguous decoding maps (Fig. 4B; Appendix Fig. S2) and more faithful reconstructions of mutant embryo pair-rule expression (Fig. 4C; Appendix Fig. S3). Quantitatively, neighborhood-informed decoding significantly reduces the posterior position standard deviation in all one-perturbed maternal signal mutants (Fig. 4D,E; Appendix Fig. S2).

Together, these results suggest that neighborhood-informed position decoding may better characterize the underlying mechanism mapping gap gene expression to positional readout in the form of pair-rule gene expression.

### Neighborhood-informed positional error in gastruloids and the developing neural tube

So far, we have only considered the setting of neighborhood-informed positional information in *Drosophila* embryos; however, this concept can be generalized to other developmental contexts as well. We consider two additional developmental systems which were previously analyzed (Merle et al, 2024; Zagorski et al, 2017) in the context of positional information along a single developmental axis of interest (either the anterior-posterior (AP) or the dorsal–ventral (DV) axis): gastruloids derived from mouse embryonic stem cells (Merle et al, 2024), and the developing neural tube in mice (Zagorski et al, 2017). Both, similarly to *Drosophila* embryos, exhibit a set of genes that determine reproducible patterning across individuals (Merle et al, 2024; Zagorski et al, 2017). These expressions of genes key to the development of both organisms, {*Bra, Cdx2, FoxC1, Sox2*} for the gastruloids and {*Shh, BMP*} for the neural tube (“Methods”), were immunofluorescently stained and imaged at precisely measured developmental stages. Expression levels were then estimated by averaging the fluorescent intensity perpendicular to the axis of interest (AP for gastruloids and DV for the neural tube). In contrast with the data collected for the *Drosophila* embryos (Petkova et al, 2019), the staining of genes in the gastruloids and neural tube systems was not done in parallel, and measurements are averaged over many cells perpendicular to the patterning axis (“Methods”).

**Fig. 5:**
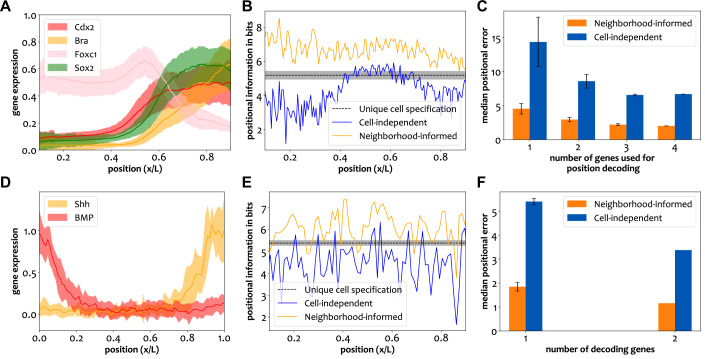
Neighborhood-informed positional information in gastruloids and the developing neural tube. (**A**) Average gene expressions (solid lines) and their standard deviations (shaded) of the four germ-layer markers: *Bra*, *Cdx2*, *FoxC1*, and *Sox2* along the AP axis of the gastruloids. (**B**) The positional information, in bits, along the AP axis of gastruloids using a neighborhood-informed decoder (orange) versus cell-independent decoding (blue). The dashed line indicates the amount of information required to uniquely specify cell location along the AP axis for the average gastruloid. (**C**) The median of the positional error across the AP axis of gastruloids (*n*_Bra_ = 48, *n*_Cdx2_ = 44, *n*_FoxC1_ = 46, *n*_Sox2_ = 138), grouped by the number of genes used for decoding. Error bars represent a single standard deviation over the choice of genes used for position decoding. (**D**) Average gene expressions (solid lines) and their standard deviations (shaded) of the two signaling gradients, *Shh* and *BMP*, in the developing vertebrate neural tube. (**E**) The positional information along the DV axis of the neural tubes when using a neighborhood-informed decoder (orange) versus cell-independent decoding (blue). The dashed line indicates the amount of information required to uniquely specify cell location. (**F**) The median of the positional error across the DV axis of the neural tube (*n*_Shh_ = 17, *n*_BMP_ = 19), grouped by the number of genes used for decoding. Source data are available online for this figure.

For gastruloids, an in vitro model for mammalian development, Merle et al (2024) showed that there is precise control over patterning across the AP axis of four germ-layer markers, *Bra, Cdx2, FoxC1*, and *Sox2*. As seen in Fig. 5A, these markers span the entire AP axis. When analyzing the gastruloids in a similar fashion to the *Drosophila* embryos (see “Methods”), we again find that the amount of positional information in the cell-independent decoder is insufficient for unique cell identification. Subsequently, using the neighborhood-informed decoder significantly increases the positional information in each position along the AP axis, as can be seen in Fig. 5B. Specifically, the information needed to uniquely decode position in gastruloids is *I*_unique_ = 5.2 ± 0.2 bits, which the neighborhood-informed decoder passes with an average of *I*_NI_ = 6.8 ± 0.6 bits across the AP axis. This is in contrast to the much lower amount of information that can be extracted from the cell-independent decoder, *I*_CI_ = 4.6 ± 1.1 bits, which is not enough for unique cell specification (“Methods”). Accordingly, the average positional information across the AP axis under the neighborhood-informed decoder is significantly higher than that of the cell-independent decoder (*P* value  < 10^−3^ under a standard *t* test). This enables much more accurate predictions of cell location, as indicated by the positional error in Fig. 5C, regardless of the number of genes used for decoding; for example, the median positional error given a single gene is $\sigma \left(\hat{x}| g,{g}_{n}\right) \sim 0.001L$ and $\sigma \left(\hat{x}| g\right) \sim 0.042L$ for neighborhood-informed and cell-independent decoders, respectively.

Similar patterning occurs in the developing vertebrate neural tube, where Sonic hedgehog (*Shh*) and bone morphogenic proteins (*BMP*) form signaling gradients across the dorsal–ventral (DV) axis, as shown in Fig. 5D. Zagorski et al (2017) showed that these antiparallel gradients can form the basis for more precise patterning of neural progenitor identities across the DV axis. Again, we find that the positional information encoded in local environments is larger than when considering the cell-independent decoder, as shown in Fig. 5E. The amount of positional information when informed by the neighborhood, *I*_NI_ = 6.1 ± 0.7 bits, is enough to uniquely specify cell positions along 89% of the DV axis, where *I*_unique_ = 5.4 ± 0.1 bits (“Methods”). In this case, again, the information that can be extracted by the cell-independent decoder is below the amount needed to uniquely specify location along 83% of the DV axis (Fig. 5E), with an average of *I*_CI_ = 4.5 ± 0.9 bits across the DV axis. Moreover, the positional error of the neighborhood-informed decoder is significantly lower than the cell-independent decoder (*P* value  < 10^−3^ under a standard *t* test), as shown in Fig. 5F; for example, the mean positional error given a single gene is $\sigma \left(\hat{x}| g,{g}_{n}\right) \sim 0.023L$ and $\sigma \left(\hat{x}| g\right) \sim 0.1L$ for the neighborhood-informed and the cell-independent decoders, respectively.

Across distinct developmental contexts, the amount of positional information to be extracted from local neighborhoods is substantially larger than in the cell-independent scenario.

## Discussion

In many contexts, cells acquire distinct position-dependent identities early in embryonic development (McGough et al, 2024; Merle et al, 2024; Moris et al, 2020; Petkova et al, 2019). Information theory provides a useful method to quantify the amount of information each cell has with regard to its position. Previous works using this approach have shown that while individual cells encode a substantial portion of the information necessary for unique identity specification, the embryo as a whole encodes the remainder (McGough et al, 2024). However, it remained unclear whether such information is biologically accessible to each individual cell.

To account for the gap in our understanding of unique cell identification, we looked into positional information embedded in the local environment of cells. This direction is inspired by a growing body of work showing that local cellular neighborhood can substantially impact cell fate (Adler et al, 2023; Bich et al, 2019; Bove et al, 2017). We mathematically modeled these local influences and showed that they can provide additional positional information, enough to determine cell positions uniquely in realistic settings.

We further corroborated our theoretical results through empirical observations in *Drosophila* embryos, gastruloids, and the developing neural tube. Throughout the different developmental contexts, we find that the positional information gap is closed along the Drosophila and gastruloid AP axis and along 89% of the DV axis in the neural tube when using the minimal-variance position estimator that incorporates neighboring expression levels. Furthermore, we showed that integration of gene expression levels in the local neighborhood reduces ambiguity in the predicted position. For *Drosophila* embryos, this lower ambiguity then improves the prediction of pair-rule gene expression patterns downstream.

To test the extent to which positional readout is encoded in local gap gene expression in *Drosophila*, we analyzed mutant embryos with disrupted maternal signals. Although these embryos have altered gap gene expression and pair-rule stripe patterns, local gap gene expression levels can be used to predict cell position with significantly reduced ambiguity. We believe that the improvements are moderate in mutant backgrounds, because there may be additional factors that influence predicted pair-rule expressions. These findings suggest that positional information alone, given a wild-type optimal decoder, may not suffice as a model for a complete understanding of decoding under disrupted conditions, and can be further adjusted in future studies.

As we have demonstrated, the positional information required for unique cell specification exists within cellular microenvironments. However, our analysis cannot on its own reveal whether spatial patterns were already inherent to the cells at the time the data was collected, or the neighborhood enables cells to be further localized, which helps the development of patterns. Perturbation experiments targeting cell–cell interactions during development are potential future steps towards distinguishing between the two scenarios.

Multiple direct and indirect mechanisms exist for the ways in which the state and fate of cells can be affected by their microenvironment, including sensing through direct cell–cell interactions (Su et al, 2024), long-range interactions by diffusing molecules (Armingol et al, 2021), the alteration of physical properties (e.g., membrane elasticity) of the cell (Heisenberg and Bellaïche, 2013; Lecuit et al, 2011), or the expression of specific adhesive molecules (Tsai et al, 2022, 2020). In this work, we focused on showing that information existing in a cell’s microenvironment is *sufficient* for accurate estimation of position, via information-theoretic considerations that are independent of the specific mechanism through which such information might be shared between neighbors. Thus, our results do not imply a causal relationship related to the potential mechanism which relays information between neighboring cells and are independent of the exact workings of such a mechanism. Crucially, this work, consistently with previous works (McGough et al, 2024), has shown that gene expression of cells by themselves lacks the amount of information needed to uniquely specify their location, which contrasts empirical evidence for developing *Drosophila* embryos (Dubuis et al, 2013a). Identifying the context-specific mechanisms or signaling pathways by which information might be transferred, for the type of neighborhood-informed decoder we describe above is an important future step. Alternatively, prior information on the mechanistic implementation of intercellular information transfer could be used for more extensive analysis of positional information.

In this work, we studied systems where uniquely specifying positional information is feasible and vital for development. Yet, such high-resolution specification of position by cells may not be feasible, or in fact, required for multicellular function in biological contexts other than those explored in this study. An interesting future direction is to explore when, and to what extent, different levels of precision in positional decoding are needed for biological function, and in particular, the connection between the sensitivity of different tissues to positional information, and how this might relate to multicellular dysfunction.

Our current framework requires that the available data comply with a set of requirements. Specifically, our positional information analysis was performed on a single spatial axis, assumed to be aligned between individuals (potentially following pre-processing of the data), and there is sufficient data for the estimation of the covariance matrices used in the calculation of the positional error. As such, in this study we focused on three developmental settings where such data is available, of high quality, and includes a sufficient number of replicates. This study lays the groundwork for studying the effects of local neighborhoods on positional information in more complex systems. Future research could extend this methodology to multidimensional contexts and more challenging, yet widely produced and accessible data types, such as spatial transcriptomics. Potential extensions of our approach to such settings could include modeling neighborhoods through a graph-based approach, which could generalize definitions of neighboring positions beyond spatial proximity. Such extensions have the potential of advancing our understanding of how interactions, whether direct or indirect, contribute to cellular decision-making and identity.

## Methods

**Table:** Reagents and tools table

Reagent/resource	Reference or source	Identifier or catalog number
**Experimental models**		
**Recombinant DNA**		
**Antibodies**		
**Oligonucleotides and other sequence-based reagents**		
**Chemicals, enzymes, and other reagents**		
**Software**		
Python 3.11	https://www.python.org/	
SciPy 1.16	10.1038/s41592-019-0686-2	
Matplotlib 3.10	10.1109/MCSE.2007.55	
**Other**		

### Description of *Drosophila* data

The empirical analyses of *Drosophila* embryos were based on the data collected by Petkova et al (2019) and is included in their Supplementary Material. For completeness, below we shortly describe the experimental setup.

WT *Drosophila* embryos were immunofluorescently stained against the four trunk gap genes, *Gt*, *Kr*, *Kni*, and *Hb* simultaneously. Two sets of embryos were collected during nuclear cycle 14. *n*_test_ = 38 embryos were collected in the 40–44 min time window, while ${n}_{{{{\rm{estim}}}}}=254$ were collected in the 38–48 min time window. We used the ${n}_{{{{\rm{estim}}}}}=254$ embryos from the 38–48 min window in order to compute all statistics needed for the estimation of positional information, while the calculation of the positional information itself was carried out on the *n*_test_ = 38 embryos collected in the narrower 40–44 min time window. When estimating and testing our hypotheses on the same data, we saw very similar overall behavior (as shown in Appendix Fig. S4). Analysis of WT pair-rule gene expression was conducted on *n*_PR_ = 34 embryos, in the 45–55 min time window, simultaneously stained for the *eve*, *prd*, and *run* pair-rule genes. The embryo fixation, antibody staining, and imaging are as described by Dubuis et al (2013a). The profile extraction similarly follows Dubuis et al (2013a), where the fluorescence intensity is averaged inside a sliding window of the size of a nucleus, and the positions of the center of the window are recorded.

Previous works have shown that the expression of both gap and pair-rule genes are highly reproducible (Dubuis et al, 2013a, 2013b; Petkova et al, 2014, 2019; Tkačik et al, 2015). In particular, the variance in the position of the peaks of pair-rule genes is smaller than the internuclear spacing (Dubuis et al, 2013b), demonstrating the level of reproducibility in pair-rule gene expression, paired with the experimental reproducibility and resolution of the data acquisition process developed in previous works (Dubuis et al, 2013a, 2013b; Petkova et al, 2019). Furthermore, the fluctuations of both gap and pair-rule genes are approximately Gaussian at each position along the AP axis (Dubuis et al, 2013b; McGough et al, 2024), which corresponds with modeling assumptions done in our work as well as previous works (Merle et al, 2024; Petkova et al, 2019; Tkačik et al, 2015).

Quantitatively, the coefficient of variation (CV, i.e., standard deviation over mean) of the expression of gap genes across the different embryos, averaged along the AP axis, was: cv_Kni_ = 0.15 ± 0.06 for *Kni*, cv_Kr_ = 0.19 ± 0.03 for *Kr*, cv_Gt_ = 0.18 ± 0.07 for *Gt*, and cv_Hb_ = 0.15 ± 0.04 for *Hb*. The CV at the local maxima of these spatial gene expressions is 0.13 ± 0.01. These statistics indicate that the standard deviation of gene expression is much lower than the expression levels themselves, implying low variability between replicates. For pair-rule genes, the corresponding CVs are: cv_Eve_ = 0.3 ± 0.1 for *Eve*, cv_Run_ = 0.2 ± 0.1 for *Run*, and cv_Prd_ = 0.4 ± 0.2 for *Prd*. These CVs, slightly higher than for gap genes, reflect low variability across replicates as well, as visually apparent from Fig. 3B. The CV of pair-rule genes at local maxima of the spatial expression, is 0.31 ± 0.04 for *Eve*, 0.33 ± 0.03 for *Run*, and 0.42 ± 0.09 for *Prd*.

Mutant *Drosophila* embryos were created by perturbing the maternal signals Bicoid (*Bcd*), Nanos (*Nos*), and Torso-like (*Tsl*). Specifically, six mutant backgrounds were created by perturbing either one or two of these signals. We use the following mutant backgrounds: *etsl*^4^ (TOR-), *bcd*^*E*1^ (BCD-), *osk*^166^ (NOS-), *bcd*^*E*2^*osk*^166^ (TOR+), *nos*^*BN*^*tsl*^1^ (BCD+), and *bcd*^*E*1^*etsl*^1^ (NOS+). Individual profiles were scaled as described in previous work (Dubuis et al, 2013a; Gregor et al, 2007). For our analysis, we consider the gap gene expression in mutant backgrounds at the 38–48  min time window for 40 *etsl*^4^ embryos, 20 *bcd*^*E*1^ embryos, 28 *osk*^166^ embryos, 15 *bcd*^*E*2^*osk*^166^ embryos, 19 *nos*^*BN*^*tsl*^1^ embryos, and 31 *bcd*^*E*1^*etsl*^1^ embryos. For pair-rule gene expression in mutant backgrounds, we consider the 45–55 min time window for 14 *etsl*^4^ embryos, 12 *bcd*^*E*1^ embryos, 11 *osk*^166^ embryos, 17 *bcd*^*E*2^*nos*^*BN*^ embryos, 32 *nos*^*BN*^*tsl*^1^ embryos, and 20 *bcd*^*E*1^*etsl*^1^ embryos.

These mutant embryos act as a strong ablation to verify our approach. Generally, the expression of gap and pair-rule genes has similar levels of variation between replicates when compared to their WT counterparts; Gap and pair-rule genes in WT embryos have a CV of ${{{{\rm{cv}}}}}_{{{{\rm{WT}}}}}^{gap}=0.16\pm 0.02$ and ${{{{\rm{cv}}}}}_{{{{\rm{WT}}}}}^{pair}=0.28\pm 0.07$, respectively. The *osk* mutant background, analyzed in Fig. 4, has comparable values of ${{{{\rm{cv}}}}}_{osk}^{gap}=0.20\pm 0.07$ and ${{{{\rm{cv}}}}}_{osk}^{pair}=0.23\pm 0.07$. Furthermore, expression levels of gap genes in these mutant embryos overlap with those of WT embryos along most of the AP axis Petkova et al (2019). To demonstrate this, the authors Petkova et al (2019) considered deviations from average expressions per position, weighted by the standard deviation of the expression per gene and per location, and showed that for the single-knockout mutants (*etsl*^4^, *bcd*^*E*1^ and *osk*^166^ embryos) the range of expressions of gap genes in the mutants falls within the 98% percentile of WT embryos along most of the AP axis. In these embryos, we find that the neighborhood-informed positional decoding more accurately predicts downstream pair-rule gene expression when compared to the cell-independent positional decoder (Fig. 4; Appendix Fig. S2). For double-knockout (*bcd*^*E*2^*nos*^*BN*^, *nos*^*BN*^*tsl*^1^, and *bcd*^*E*1^*etsl*^1^) embryos, the expression of gap genes was further removed from the range of their expressions in WT embryos as opposed to the single-knockout mutants, crossing the 98% percentile of WT expressions along large swathes of the AP axis (Petkova et al, 2019). We reason that this underlies the more modest improvements in the prediction of pair-rule gene expression from gap genes in double-knockout, relative to single-knockout mutants, under the neighborhood-informed positional decoding framework in comparison to the cell-independent positional decoding (shown in Appendix Fig. S2d–f).

Using image processing software, McGough et al (2024) counted *N* = 72 ± 3 nuclei across the middle 80% of the AP axis. This then corresponds to *N* = 90 ± 4 nuclei across the full AP axis, if the nuclei are assumed to be uniformly distributed across the AP axis. For our calculation of the information required to uniquely specify cell location, we used *N* = 90 ± 4 nuclei.

### Description of the gastruloids data

The analysis of gastruloids was carried out on the data collected by Merle et al (2024), which is included in their Supplementary Material. The gastruloids were derived from mouse embryonic stem cells, with an initial seed of ${\bar{N}_{0}}=300$ cells. Four germ-layer markers, previously observed to have pivotal roles in the differentiation of tissue progenitors along the AP axis, were immunofluorescently stained and confocally imaged, 120 h after seeding. Two-dimensional maximum projections were then chosen from the confocal image, from which one-dimensional intensity profiles were extracted along the gastruloid’s midline, using standard image processing approaches. In practice, these 1D profiles are the average of the expression levels perpendicular to the midline, which was segmented into *n*_*B*_ = 200 bins. Full details for all processes can be found in (Merle et al, 2024).

In contrast with the *Drosophila* embryos from “Description of *Drosophila* data”, the gastruloids were not jointly stained for all markers. Instead, *n*_Bra_ = 48 gastruloids were stained for both *Bra* and *Sox2*, *n*_Cdx2_ = 44 gastruloids were stained for both *Cdx2* and *Sox2*, and finally another *n*_FoxC1_ = 46 were stained for both *Foxc3* and *Sox2*. Thus, when analyzing the positional information, we considered only the variance of each gene when estimating the gene-covariance matrix needed for Eq. (5). Calculating the positional information with this covariance constitutes a lower bound on the total information available at the same location. Otherwise, all other calculations are carried out exactly as for the *Drosophila* embryos.

The focus of Merle et al (2024) on the set of genes described above was guided by their well-documented importance toward patterning along the AP axis during development (Blassberg et al, 2022; Mittnenzweig et al, 2021; Neijts et al, 2014). As in *Drosophila* embryos, we calculated the coefficient of variation (CV) of gene expression along the AP axis of the gastruloids and found that the variation due to replicates was relatively low: cv_Cdx2_ = 0.4 ± 0.2 for *Cdx2*, cv_Bra_ = 0.8 ± 0.6 for *Bra*, cv_Foxc1_ = 0.3 ± 0.2 for *FoxC1*, and cv_Sox2_ = 0.5 ± 0.4 for *Sox2*.

To estimate *I*_unique_, the length was chosen as the average length of the gastruloid’s midline *L* = 494 ± 52 μm, and the number of cells was estimated as *N* = 〈*L*〉/〈*d*_*c*_〉 where *d*_*c*_ = 13.5 ± 0.8 μm is the diameter of a cell.

### Description of the developing neural tube data

Expression profiles along the dorsal–ventral axis of the developing neural tube in mice were provided by direct correspondence with the authors of Zagorski et al (2017). Transverse brachial sections were collected from growing mice at 35h, and immunofluorescently stained against Sonic hedgehog (*Shh*) and bone morphogenetic proteins (*BMP*s), which are antiparallel signaling gradients along the dorsal–ventral (DV) axis of the developing vertebrate neural tube. Previous work has hypothesized that the precision of the DV pattern of neural progenitor identities depends to an extent on *Shh* and *BMP*. As in “Description of the gastruloids data”, intensity profiles were collected as the average intensity perpendicular to the DV axis, segmented into 90 bins. Further details can be found in (Zagorski et al, 2017).

Once again, as in “Description of the gastruloids data”, the neural tubes were not jointly stained for *Shh* and *BMP*. Instead, *n*_Shh_ = 17 samples were stained against *Shh*, and *n*_BMP_ = 19 samples were stained against *BMP*. As such, for the covariance matrix in Eq. (5) only the diagonal of the matrix was set to be non-zero. The same smoothing as in “Description of the gastruloids data” for the derivative of average gene expression was used. The length of the collected neural tubes was *L* = 204 μm and *d*_c_ = 4.9 ± 0.4 μm. These data were slightly more variable between replicates when compared to the *Drosophila* embryos and gastruloids, with cv_Shh_ = 0.91 ± 0.34 for *Shh*, and cv_Shh_ = 0.87 ± 0.29 for *BMP*.

### Decoding the position of the neighboring gene expression

We will study the case where *g*_*n*_ represent the gene expressions at *x*’s adjacent positions, that is ${g}_{n}={\left[g(x-1),g(x+1)\right]}^{T}$. *g*_*n*_ ∈ **R**^2*d*^ and *g*_env_ = [*g*, *g*_*n*_] ∈ **R**^3*d*^ where *d* is the number of genes considered. Following previous work (Dubuis et al, 2013b; Petkova et al, 2019), we will assume that the fluctuations in expression at each position along the AP axis are Gaussian, so that:

9 $${{{\rm{P}}}}\left({g}_{{{{\rm{env}}}}}| \hat{x}\right)=\frac{1}{\sqrt{{(2\pi )}^{k}\parallel {C}_{{{{\rm{env}}}}}\parallel }}{e}^{-{\chi }_{k}^{2}({g}_{{{{\rm{env}}}}},\hat{x})/2}$$

is the likelihood of the expression pattern *g*_env_ at position $\hat{x}$, and:

10 $${\chi }_{k}^{2}({g}_{{{{\rm{env}}}}},\hat{x})={\sum }_{\ell ,j=1}^{k}{\left[{g}_{{{{\rm{env}}}}}-{\overline{g}}_{{{{\rm{env}}}}}\right]}_{\ell }{[{C}_{{{{\rm{env}}}}}^{-1}]}_{\ell j}{\left[{g}_{{{{\rm{env}}}}}-{\overline{g}}_{{{{\rm{env}}}}}\right]}_{j}$$

measures how similar the gene expression patterns in one embryo at position *x*, *g*_env_, is to the mean expression over embryos at the same position, ${\overline{g}}_{{{{\rm{env}}}}}$. *C*_env_ ∈ **R**^3*d*×3*d*^ is the covariance matrix of *g*_env_.

Using Bayes’ law we can determine the posterior distribution over positions $\hat{x}$ given the local gene expression, *g*_env_:

11 $$P(\hat{x}| {g}_{{{{\rm{env}}}}})=\frac{1}{Z({g}_{{{{\rm{env}}}}})}P({g}_{{{{\rm{env}}}}}| \hat{x}){P}_{x}(\hat{x})$$

Here, ${P}_{x}(\hat{x})$ is the prior distribution that a cell is at position $\hat{x}$, which, given no additional information, will be assumed to be uniform. *Z*(*g*_env_) serves to normalize the distribution and is independent of the position $\hat{x}$.

### Decoding map representation

The decoding map is a probabilistic representation of the predicted positions relative to the ground-truth positions, which is schematically described in Fig. 1A. The *x* axis represents the ground-truth positions along the anterior-posterior axis, *x*. The *y* axis represents the predicted positions $\hat{x}$. $x,\hat{x}\in [1,2,\cdots L]$ where *L* is the number of positions along the AP axis. Therefore, each column represents the posterior distributions over positions given gap gene expression patterns, namely $P(\hat{x}| {g}_{{{{\rm{env}}}}})$ when decoding in a neighborhood-informed fashion, or $P(\hat{x}| g)$, when decoding in a cell-independent manner. The decoding map is calculated per embryo, and we present the sum of the decoding maps over all embryos in logarithm scale, to retain a meaningful dynamic range.

### Quantifying the error in the prediction of the location

Here, we aim to quantify the accuracy of position prediction. To do so, we first compute the maximum a-posteriori (MAP) of the distributions $P(\hat{x}| {g}_{{{{\rm{env}}}}})$ (neighborhood-informed) and $P(\hat{x}| g)$ (cell-independent), as the predicted location given gene expression levels at the neighborhood environment of *x* or at the position *x*, respectively:

12 $${\hat{x}}_{{{{\rm{NI}}}}}^{(\alpha )}({g}_{{{{\rm{env}}}}}^{(\alpha )})=\arg {\max }_{\hat{x}}P\left(\hat{x}| {g}_{{{{\rm{env}}}}}^{(\alpha )}\right)$$

where ${g}_{{{{\rm{env}}}}}^{(\alpha )}$ is the gene expression of cell *x* and its adjacent positions in embryo *α*. Together, ${\hat{x}}_{{{{\rm{NI}}}}}^{(\alpha )}({g}_{{{{\rm{env}}}}}^{(\alpha )})$ can be considered as the neighborhood-informed (NI) position prediction. Analogously, for cell-independent decoding, the predicted position is given by:

13 $${\hat{x}}_{{{{\rm{CI}}}}}^{(\alpha )}({g}^{(\alpha )})=\arg {\max }_{\hat{x}}P\left(\hat{x}| {g}^{(\alpha )}\right)$$

For each embryo *α*, we then calculate a vector of the MAP positions at each position along the AP axis:

14 $${\hat{x}}_{{{{\rm{NI}}}}}^{(\alpha )}=\left[{\hat{x}}_{{{{\rm{NI}}}}}^{(\alpha )}({g}_{{{{\rm{env}}}}}^{(\alpha )}(1)),\cdots \,,{\hat{x}}_{{{{\rm{NI}}}}}^{(\alpha )}({g}_{{{{\rm{env}}}}}^{(\alpha )}(L))\right]\in {{\mathbb{R}}}^{L}$$

where *L* is the length of the embryo. The positions 1, ⋯  , *L* are the assumed ground-truth positions, which we also arrange in a vector ${x}_{{{{\rm{gt}}}}}=\left[1,\cdots \,,L\right]\in {{\mathbb{R}}}^{L}$. We define the analogous MAP vector for cell-independent decoding, ${\hat{x}}_{{{{\rm{CI}}}}}^{(\alpha )}\in {{\mathbb{R}}}^{L}$.

To quantitatively measure the positional prediction error, we compute the mean absolute difference between the ground-truth position and the MAP of the posterior distribution of predicted position over all embryos and over all positions. Specifically, the neighbor-informed positional prediction error is:

15 $$\,{{{\rm{pos}}}} {\mbox{-}} {{{\rm{error}}}}\,({{{\rm{NI}}}})=\frac{1}{M\cdot L}{\sum }_{\alpha =1}^{M}{\left\Vert {\hat{x}}_{{{{\rm{NI}}}}}^{(\alpha )}-{x}_{{{{\rm{gt}}}}}\right\Vert }_{1}$$

and the cell-independent positional prediction error is:

16 $$\,{{{\rm{pos}}}} {\mbox{-}} {{{\rm{error}}}}\,({{{\rm{CI}}}})=\frac{1}{M\cdot L}{\sum }_{\alpha =1}^{M}{\left\Vert {\hat{x}}_{{{{\rm{CI}}}}}^{(\alpha )}-{x}_{{{{\rm{gt}}}}}\right\Vert }_{1}$$

### Estimation of positional error

The positional error as defined in Eq. (5) is explicitly calculated as (Dubuis et al, 2013b):

17 $$\frac{1}{{\sigma }^{2}(\hat{x}| g)}={\Sigma }_{i,j=1}^{4}{\left[\frac{d{\overline{g}}_{i}(x)}{dx}{(C{(x)}^{-1})}_{ij}\frac{d{\overline{g}}_{j}(x)}{dx}\right]}_{x=\hat{x}({g}_{k})}$$

where *g*_*i*_ represents the expression of gap gene *i* (the gap genes include {Kr, Kni, Hb, Gt}), $\frac{d{\overline{g}}_{i}(x)}{dx}$ is the derivative of the mean expression over embryos of *g*_*i*_ at position *x*, and *C*(*x*) is the covariance matrix of the gene expression of the four gap genes at position *x*.

When decoding position with neighboring gene expression, the positional error ${\sigma }^{2}(\hat{x}| {g}_{{{{\rm{env}}}}}(x))$ integrates the expression at the adjacent right and left positions, explicitly:

18 $$	\frac{1}{{\sigma }^{2}(\hat{x}| {g}_{{{{\rm{env}}}}}(x))} \\ 	 = {\Sigma }_{i,j = 1}^{4}{\left[\frac{d[{ {\bar{g}} }_{i}(x),{ {\bar{g}} }_{i}(x-1),{ {\bar{g}} }_{i}(x+1)]}{dx}{({C}_{{{{\rm{env}}}}}{(x)}^{-1})}_{ij}\frac{d[{ {\bar{g}} }_{j}(x),{ {\bar{g}} }_{j}(x-1),{ {\bar{g}} }_{j}(x+1)]}{dx}\right]}_{x=\hat{x}({g}_{k})}$$

where *C*_env_(*x*) is the covariance matrix of the gene expression of the four gap genes at positions *x*, *x* − 1, and *x* + 1.

### Estimation of the amount of positional information

The amount of positional information *I*_position_ extracted from gene expression levels at a specific location along a single patterning axis of length *L* can be estimated by (Dubuis et al, 2013b):

19 $${I}_{{{{\rm{position}}}}}={\log }_{2}L-{\log }_{2}\left(\sqrt{2\pi e}\cdot \sigma (\hat{x}| g(i))\right)$$

where $\sigma (\hat{x}| g(i))$ is the standard deviation in a position decoder’s prediction $\hat{x}$ given gene expression levels *g*(*i*) at the *i*th position. Thus, the amount of positional information is related to the position decoder.

The positional information we report for the cell-independent decoder *I*_CI_ is averaged across the length of the embryo and given by:

20 $${I}_{{{{\rm{CI}}}}}={\log }_{2}L-\frac{1}{N}{\sum }_{i=1}^{N}{\log }_{2}\left(\sqrt{2\pi e}\cdot \sigma \left(\hat{x}| g(i)\right)\right)$$

where *g*(*i*) denotes the gene expression levels at position *i*. The standard deviation of the decoder is estimated according to Eq. (17).

The calculation carried out for the neighborhood-informed positional information is analogous, only using the neighborhood-informed decoder:

21 $${I}_{{{{\rm{NI}}}}}={\log }_{2}L-\frac{1}{N}{\sum }_{i=1}^{N}{\log }_{2}\left(\sqrt{2\pi e}\cdot \sigma \left(\hat{x}| {g}_{{{{\rm{env}}}}}(i)\right)\right)$$

where *g*_env_(*i*) are the gene expression levels in the microenvironment of position *i*, given by: *g*_env_(*i*) = {*g*(*i* − 1), *g*(*i*), *g*(*i* + 1)}. The variance of the decoder for each position in this case is estimated according to Eq. (5).

Throughout our work, we compare the above notions of positional information to the amount of information required to uniquely specify location. Following McGough et al (2024), this amount is given by:

22 $${I}_{{{{\rm{unique}}}}}={\log }_{2}N$$

where *N* is the number of cells along the patterning axis of the embryo.

### Pair-rule gene expression comparisons and reconstruction

Using the position decoder, we reconstructed the predicted wild-type pair-rule gene expression profiles along the AP axis of the embryo. This reconstruction of expression can be compared to the wild-type’s mean pair-rule gene expression profile across the AP axis, weighted by the noise in expression at each position. Formally, the expected expression of pair-rule gene *g*_*k*_ based on a neighborhood-informed decoding is:

23 $${\mathbb{E}}[{ {\bar{g}} }_{k}(\hat{x})| {g}_{{{{\rm{env}}}}}(x)]= {\Sigma }_{\hat{x}}p\left(\hat{x}| {g}_{{{{\rm{env}}}}}(x)\right)\cdot { {\bar{g}} }_{k}(\hat{x})$$

The analogous expression for a cell-independent position decoding:

24 $${\mathbb{E}}[{ {\bar{g}} }_{k}(\hat{x})| g(x)]= {\Sigma }_{\hat{x}}p(\hat{x}| g(x)){ {\bar{g}} }_{k}(\hat{x})$$

Where ${ {\bar{g}} }_{k}(\hat{x})$ is the mean expression of pair-rule gene *k* in wild-type embryos at position $\hat{x}$.

The reconstruction error is then the absolute error between the ground-truth and predicted pair-rule gene expression. The neighborhood-informed reconstruction error is given by:

25 $$\,{{{\rm{recon}}}} {\mbox{-}} {{{\rm{error}}}}\,{(NI)}_{k}=\frac{1}{M\cdot L}{\sum }_{\alpha =1}^{M}{\sum }_{i=1}^{L}\left|\frac{{\mathbb{E}}\left[{\overline{g}}_{k}(\hat{x})| {g}_{{{{\rm{env}}}}}^{(\alpha )}(i)\right]-{\overline{g}}_{k}(i)}{std({g}_{k}(i))}\right|$$

where *M* is the number of embryos, *L* is the number of positions along the AP axis, and *std*(*g*_*k*_(*i*)) is the standard deviation across embryos in gene expression of pair-rule *g*_*k*_ at position *i*. The reconstruction error for the cell-independent decoder is calculated analogously.

For mutant embryos, the ground-truth expression of pair-rule genes, ${\overline{g}}_{k}(i)$, is taken for the mutant embryos and not the wild-type; however, the reconstruction is done using the ${ {\bar{g}} }_{k}(\hat{x})$ as in the wild-type case (Eqs. (23) and (24)).

## Graphics

Synopsis, Figs. 2, and 3 graphics were created with BioRender.com.

## Supplementary information

Peer Review File

Appendix

Source data Fig. 1

Source data Fig. 2

Source data Fig. 3

Source data Fig. 4

Source data Fig. 5

## Data Availability

The computer code produced in this study is available on GitHub (https://github.com/nitzanlab/Neighborhood-Informed-Positional-Information). The source data of this paper are collected in the following database record: biostudies:S-SCDT-10_1038-S44320-026-00211-y.
