# Supplementary material for: Neighborhood-informed positional information for precise cell identity specification
Source: Mol Syst Biol. 2026 May 5;22(7):1118–31. doi: 10.1038/s44320-026-00211-y (PMC13328375; doi:10.1038/s44320-026-00211-y)
Supplement: Supplementary file 2 — Appendix [file 44320_2026_211_MOESM2_ESM.pdf]

Appendix for Neighborhood-Informed Positional Information for Precise Cell Identity Specification

Contents

1 Introduction to information-theoretic analysis of positional information 2

1.1 Positional information in bits 2

1.2 A gap in positional information 3

2 Pairwise correlations close the information gap 4

3 Decoding with neighboring gene information strictly decreases position error 6

4 Explicit form of information gap under Gaussian assumptions 8

5 Appendix figures 10

1 Introduction to information-theoretic analysis of positional information

In recent years, a growing body of work (Dubuis et al. 2013b; Tkačik et al. 2015; Petkova et al. 2019; McGough et al. 2024) has been dedicated to utilizing information-theoretic methodologies to better understand whether cells have potential access to sufficient information to accurately determine their position in space, particularly during development. Intuitively, such cellular spatial orientation could facilitate coordination and specialization by multitudes of cells during development, where position could potentially be used as a readout to define cellular roles required to form healthy, functioning tissues and full organisms (Wolpert 1971). The core idea underlying this line of works is that it is possible to estimate quantities of information available directly from marginal signals (such as the expression of key genes) even when there is no direct access to molecular mechanisms. In this work, we build on this concept of *positional information* and extend it to a neighborhood-informed setting. Below we provide a brief introduction to key-concepts introduced in previous works. We refer to Tkačik and Gregor (2021) for a more in-depth discussion of the role of information theory in the field of positional information.

1.1 Positional information in bits

The seminal work of Dubuis et al. (2013b) laid the foundations necessary to rigorously quantify the amount of positional information available to each cell individually through an information-theoretic approach. This formulation was then utilized to estimate the amount of positional information available in a range of scenarios and organisms (Tkačik et al. 2015; McGough et al. 2024; Zagorski et al. 2017; Petkova et al. 2019; Merle et al. 2024).

To determine the amount of information a cell has regarding its position requires a manner to probe the state of the cell. In practice, we can empirically measure only a small portion of the factors effecting the state of the cell, usually given in the form of a subset of genes expressed by the cell, $g \in \mathbb{R}^d$. These gene expression observations greatly reduce the ambiguity regarding the state of the cell relative to the uncertainty in position given no information regarding gene expression. For simplicity, we will consider a one dimensional axis along an organism, in which case all cells are positioned along a single axis, denoted by $x \in \mathbb{R}$. The above means that, given a specific observation regarding gene expression levels, there is a distribution of possible positions which correspond with specific gene expression levels, which we denote by $P(x|g)$. The amount of information that can be extracted from samples from this distribution is larger than from samples of the positions given no observations regarding gene expression, $P(x)$, which is taken to be a uniform distribution along the whole positional axis. The difference between these distributions, $P(x|g)$ and $P(x)$, can be leveraged to determine the amount of positional information contained in g.

Qualitatively, the amount of positional information made available by the expression of the genes g is defined by the reduction of ambiguity regarding position in $P(x|g)$ relative to $P(x)$. Formally, the ambiguity of a distribution is defined through

its *entropy*:

$$H[P(x|g)] = -\mathbb{E}_{x|g}[\log_2 P(x|g)] \tag{1}$$

$$H[P(x)] = -\mathbb{E}_x[\log_2 P(x)] = \log_2 L \tag{2}$$

where L is the length of the developmental axis. The $\log_2 L$ in Equation (2) is due to the assumption that the distribution over positions is a priori uniform along the full developmental axis. The average information afforded by g is then equal to the average difference between the above entropy terms:

$$I_{\text{position}} = \mathbb{E}_g[H[P(x)] - H[P(x|g)]] \tag{3}$$

$P(x|g)$ is well approximated by a normal distribution Dubuis et al. (2013b); McGough et al. (2024), in which case the amount of positional information takes the following, explicit form:

$$I_{\text{position}} = \log_2 L - \log_2\left(\sqrt{2\pi e} \cdot \sigma(x|g)\right) \tag{4}$$

where $\sigma(x|g)$ is the standard deviation of the conditional distribution $P(x|g)$.

The above positional information, I_{position}, is measured in *bits*. The number of bits reflects the accuracy with which gene expression can determine location, in terms of halvings of the developmental axis; for instance, when $I_{\text{position}} = 1$ bits, then the information gained from having access to gene expression levels could allow a cell to determine whether it is in the first half of the axis or the second. When $I_{\text{position}} = 2$ bits, the gene expression levels carry enough information to uniquely specify location to each quarter of the axis, and so on. This perspective of positional information enables us to estimate the accuracy with which it is possible to find a cell along the development axis, and in this way can also be understood through the scope of probabilistic "decoding" the position x from the observed genes, g (Petkova et al. 2019).

The amount of positional information estimated in this manner is independent of the particular mechanism which brought forth the particular expression of genes in the cell. This methodology is limited by the available data, and is limited to the analysis of positional information accessible via the set of genes that can be empirically measured, which in turn need to be measured with high spatial resolution across multiple replicates.

1.2 A gap in positional information

As part of the developmental process of *Drosophila* embryos, pair-rule genes are expressed in a precise striping pattern along the AP axis of the embryo. It was empirically observed that the positions of key landmarks along the AP axis, formed by local maxima in the expression of pair-rule genes, exhibit a lower variability than the internuclear distance (Dubuis et al. 2013a; McGough et al. 2024). This potentially implies that in such developmental contexts cells can uniquely specify their locations. In such scenarios, we should expect that $I_{\text{position}} \geq \log_2(N)$ where N is the number of nuclei along the patterning axis, which means that the genes contain enough information to

divide the axis to cell-sized bins. As mentioned above, this is reportedly the case for *gap* genes in key developmental stages of *Drosophila* embryos (Dubuis et al. 2013a; McGough et al. 2024).

McGough et al. (2024) demonstrated that the positional information inherent to gap genes in *Drosophila* embryos falls below the bound required for uniquely specifying cell positions. In other words, there is a gap between the information required to uniquely specify cell positions and the amount of positional information afforded by gap genes, which does not align with observations regarding the low amount of variability of positions of the positions of the striping patterns along the AP axis of *Drosophila* embryos (Dubuis et al. 2013a).

2 Pairwise correlations close the information gap

In previous work, McGough et al. (2024) modeled the correlations in the predicted positions as:

$$\mathrm{corr}\left[\hat{x}_n, \hat{x}_{n-\Delta x}\right] = e^{-\frac{L}{\xi N}\Delta x} \tag{5}$$

where Δx is the distance between two nuclei along the AP axis, $\hat{x}_n$ is the predicted position for the n-th nucleus and $\xi > 0$ is the correlation length across the AP axis of length L and N nuclei. McGough et al. (2024) further showed that the distribution of $\hat{x}_n$ for all n is approximately Gaussian, in which case the joint distribution for each pair $p\left(\hat{x}_{n+1}, \hat{x}_n\right)$ is a bivariate Gaussian distribution.

We will now proceed to show that one manner in which such a correlation structure can come about is if the gene expressions are Markovian along the AP axis. In other words, such correlation patterns arise when we assume that:

$$\hat{x}_{n+1}, \hat{x}_n p\left(\hat{x}_{n+1}|\hat{x}_n, \hat{x}_{n-1}, \cdots\right) = p\left(\hat{x}_{n+1}|\hat{x}_n\right) \tag{6}$$

This Markovian assumption follows the intuition that the nuclei of cells are most affected by their neighbors, and not those that are further down the AP axis. Using this Markovian we can define the long-range correlations in terms of local correlations between adjacent positions.

For positions that are equally spaced along the AP axis, the above assumptions dictate the following Markov chain:

$$p\left(\hat{x}_{n+1}|\hat{x}_n\right) = \mathcal{N}\left(\hat{x}_{n+1}| \quad a_{n+1|n}\left(\hat{x}_n - \mu_x\right) + \mu_{n+1},\ \sigma^2_{n+1|n}\right) \tag{7}$$

where $a_{n+1|n}$ is the transition of the Markov chain from $\hat{x}_n$ to $\hat{x}_{n+1}$, and $\sigma^2_{n+1|n}$ is the variance of $p\left(\hat{x}_{n+1}|\hat{x}_n\right)$. Since the joint distribution $p(\hat{x}_{n+1}, \hat{x}_n)$ is a Gaussian distribution (through the assumptions above), then both $a_{n+1|n}$ and $\sigma^2_{n+1|n}$ can be written in closed form as:

$$a_{n+1|n} = \frac{\sigma_{n+1,n}}{\sigma^2_n} \tag{8}$$

$$\sigma^2_{n+1|n} = \sigma^2_{n+1} - \frac{\sigma^2_{n+1,n}}{\sigma^2_n} \tag{9}$$

where $\sigma_{n,n+1}$ is the covariance between $\hat{x}_n$ and $\hat{x}_{n+1}$. Accordingly, σ_n^2 is the variance of $\hat{x}_n$.

In the particular case that $\mu_n = 0$, $\sigma_{n,n+1} = c$, and $\sigma_n^2 = \sigma_c^2$ for all n, then the above Markov chain describes long-range correlations in Equation (5), which we will now show. These two definitions mean that the scaling between stages of the Markov chain are the same for all n, and we can write $a_{n+1|n} = a$. Every step in the chain is then given by the distribution of the preceding element:

$$\hat{x}_{n+1} = \frac{c}{\sigma_c^2}\hat{x}_n + c\epsilon_{n+1} = a\hat{x}_n + c\epsilon_{n+1} \tag{10}$$

$$\epsilon_{x+1} \sim \mathcal{N}(0, 1) \tag{11}$$

where ϵ_{n+1} is the randomness introduced when transitioning from cell n to cell $n+1$.

We can now unroll the Markov chain using the step from Equation 10, up to Δ steps:

$$\hat{x}_n = a\hat{x}_{n-1} + c\epsilon_x \tag{12}$$

$$= a^2\hat{x}_{n-2} + ac\epsilon_{x-1} + c\epsilon_x \tag{13}$$

$$\vdots \tag{14}$$

$$= a^\Delta\hat{x}_{n-\Delta} + \sum_{i=0}^{\Delta-1} a^m c^2 \epsilon_{x-m} \tag{15}$$

This allows us to explicitly write the variance of $\hat{x}_n$ given an observation of $\hat{x}_{n-\Delta}$:

$$\mathrm{var}\left[\hat{x}_n|\hat{x}_{n-\Delta}\right] = \sum_{m=0}^{\Delta-1} a^{2m}\sigma_c^2 - \sum_{m=0}^{\Delta-1} a^{2m}\frac{c^2}{\sigma_c^2} \tag{16}$$

Using the properties of the Gaussian distribution, we can derive the joint covariance from the conditional variance $\sigma^2_{n|n-\Delta}$ together with that of the marginal variance $\sigma^2_{n-\Delta}$. Taking into account that the right hand side of Equation (16) is a telescoping sum, and substituting a, we get:

$$\mathrm{cov}\left[\hat{x}_n, \hat{x}_{n-\Delta}\right] = a^{\Delta-1}c \tag{17}$$

$$= \underbrace{\frac{c}{\sigma_c^2}\cdots\frac{c}{\sigma_c^2}}_{\Delta x-1 \text{ times}} \cdot c \tag{18}$$

$$= \frac{c^\Delta}{\sigma_c^{2(\Delta-1)}} \tag{19}$$

Finally, the correlations are given by:

$$\mathrm{corr}\left[\hat{x}_n, \hat{x}_{n-\Delta}\right] = \frac{\mathrm{cov}\left[\hat{x}_n, \hat{x}_{n-\Delta}\right]}{\sigma_c^2} \tag{20}$$

$$= \left(\frac{c}{\sigma_c^2}\right)^{\Delta x} \tag{21}$$

$$= \text{corr}\left[\hat{x}_n, \hat{x}_{n-1}\right]^{\Delta x} \tag{22}$$

In the special case where $c/\sigma_c^2 = \exp[-L/N\xi]$ for some ξ, which is a reparametrization that is always possible whenever $c > 0$, we arrive at:

$$\text{corr}[\hat{x}_n, \hat{x}_{n-\Delta}] = \exp\left[-\frac{L}{N\xi}\Delta x\right] \tag{23}$$

which is the same as Equation 5.

To conclude, with the small addition of the mild Markovian assumption atop those made in previous work (McGough et al. 2024), long-range correlations are the result of correlations between neighboring cells. As shown by McGough et al. (2024), when $\xi \geq 19.5\frac{L}{N}$, the information gap is closed. In terms of pairwise correlations, this entails that if:

$$\text{corr}\left[\hat{x}_n, \hat{x}_{n+1}\right] \geq 0.95 \tag{24}$$

for all n, then the information gap is closed.

3 Decoding with neighboring gene information strictly decreases position error

In this section we will show that the lower-bound on the amount of positional information strictly increases when adding neighboring information, as long as the correlations between the neighbors are not equal to ± 1.

We will look at the case where the gene expression of x, g, and its neighbors g_n are jointly observed. Here we assume that $g \in \mathbb{R}^d$ and $g_n \in \mathbb{R}^{n \cdot d}$ where n are the number of neighbors. A lower bound for the variance of any predictor $\hat{x}$ can be found using the Cramer-Rao bound. When only g is observed, this is equal to:

$$\frac{1}{\sigma^2(\hat{x}|g)} \leq \left(\frac{\partial \overline{g}}{\partial x}\right)^T \Sigma^{-1} \left(\frac{\partial \overline{g}}{\partial x}\right) \tag{25}$$

where $\overline{g}$ is the mean gene expression and Σ is the covariance matrix between all genes, which we will assume is independent of the position x. This is the cell-independent version of the Cramer-Rao bound on the decoder variance, to which we will relate the neighborhood-informed version.

The lower bound on the variance of the neighborhood-informed decoder will depend on the inverse covariance between g and g_n. As such, to explicitly write down the neighborhood-informed lower bound, we need to invert the covariance of g and g_n:

$$\Sigma_g^{-1} = \begin{bmatrix} \Sigma_c & \Sigma_{cn} \\ \Sigma_{cn}^T & \Sigma_n \end{bmatrix}^{-1} \tag{26}$$

$$= \begin{bmatrix} \Sigma_c^{-1} + \Sigma_c^{-1}\Sigma_{cn}D^{-1}\Sigma_{cn}^T\Sigma_c^{-1} & -\Sigma_c^{-1}\Sigma_{cn}D^{-1} \\ -D^{-1}\Sigma_{cn}^T\Sigma_c^{-1} & D^{-1} \end{bmatrix} \tag{27}$$

where Σ_c is the marginal covariance of g, Σ_n is the marginal covariance of g_n and $D = \Sigma_n - \Sigma_{cn}^T\Sigma_c^{-1}\Sigma_{cn}$.

Defining:

$$\nu_g = \begin{pmatrix} \partial\overline{g}/\partial x \\ \partial\overline{g_n}/\partial x \end{pmatrix} = \begin{pmatrix} \nu \\ \nu_n \end{pmatrix} \tag{28}$$

for ease of notation, the neighborhood-informed bound takes the following form:

$$\frac{1}{\sigma^2(\hat{x}|g, g_n)} \leq \nu_g^T\Sigma_g^{-1}\nu_g \tag{29}$$

We can now plug Equation 27 into the above to get a precise term for the bound on the variance:

$$\nu_g^T\Sigma_g^{-1}\nu_g = \nu\Sigma_c^{-1}\nu + \nu^T\Sigma_c^{-1}\Sigma_{cn}D^{-1}\Sigma_{cn}^T\Sigma_c^{-1}\nu - 2\nu^T\Sigma_c^{-1}\Sigma_{cn}D^{-1}\nu_n + \nu_n^TD^{-1}\nu_n \tag{30}$$

For ease of notation, we will define $\gamma = \Sigma_{cn}^T\Sigma_c^{-1}\nu$. Using this shorthand, the above simplifies to:

$$\nu_g^T\Sigma_g^{-1}\nu_g = \underbrace{\nu^T\Sigma_c^{-1}\nu}_{(*)} + \underbrace{(\gamma - \nu_n)^T D^{-1}(\gamma - \nu_n)}_{(\dagger)} \tag{31}$$

The $(*)$ term is exactly the same as the lower bound on the cell-independent variance, which is the RHS of Equation 25.

To show that the neighborhood-informed bound on the variance is lower than that of the cell-independent, we must show that the $(\dagger)$ term is a positive number. As long as Σ_c, Σ_g are both positive-definite, then D must also be positive-definite as it is Schur's complement of Σ_c in Σ_g. Because of this, the $(\dagger)$ term is also guaranteed to be positive. In that case:

$$\nu^T\Sigma_c^{-1}\nu + (\gamma - \nu_n)^T D^{-1}(\gamma - \nu_n) > \nu_c^T\Sigma_c^{-1}\nu_c \tag{32}$$

$$\Leftrightarrow \frac{1}{\nu^T\Sigma_c^{-1}\nu + (\gamma - \nu_n)^T D^{-1}(\gamma - \nu_n)} < \frac{1}{\nu^T\Sigma_c^{-1}\nu} \tag{33}$$

That is, the lowest possible variance $\hat{\sigma}^2(\hat{x}|g, g_n)$ that can be attained when using information from neighbors is smaller than when not using neighbors:

$$\hat{\sigma}^2(\hat{x}|g, g_n) < \hat{\sigma}^2(\hat{x}|g) \tag{34}$$

To summarize this relation, we will define:

$$\hat{\sigma}^{-2}(x|g, g_n) = \hat{\sigma}^{-2}(x|g) + (\gamma - \nu_n)^T D^{-1}(\gamma - \nu_n) \tag{35}$$

$$= \hat{\sigma}^{-2}(x|g) + \delta_{\text{neighbor}} \tag{36}$$

where $\delta_{\text{neighbor}} = (\gamma - \nu_n)^T D^{-1} (\gamma - \nu_n)$ is the added information from the neighbors.

4 Explicit form of information gap under Gaussian assumptions

In the special case when it is assumed that x, g, g_n are jointly Gaussian, a closed-form expression for the variance of the decoder, $\sigma^2(\hat{x}|g, g_n)$, can be derived. We begin by following previous work and follow the assumption that the likelihood $p(g, g_n|x)$ is a Gaussian distribution. Furthermore, we will assume that the prior over positions is also a Gaussian distribution:

$$x \sim \mathcal{N}\left(L/2, \sigma_x^2\right) \tag{37}$$

where L is the length of the embryo and σ_x^2 is the variance of this prior over the position, and will be taken to be much larger than the length of the AP axis.

The above Gaussian assumptions force a specific form on the conditional distribution $p(g, g_n|x)$, through the definition of conditional distributions of a Gaussian. In particular, the mean and covariance of this conditional distribution take the following form:

$$\mathbb{E}\left[g_i|x\right] = \overline{g_i}\left(x\right) = \frac{(x-\mu_x)}{\sigma_x^2}\text{cov}\left(x, g_i\right) + \overline{g_i} = \frac{x-\mu_x}{\sigma_x^2}\nu_i + \overline{g_i} \tag{38}$$

$$\text{cov}\left(g, g_n|x\right) = \Sigma_g - \frac{1}{\sigma_x^2}\nu\nu^T \tag{39}$$

The value ν_i here is equal to $\text{cov}\left(x, g_i\right)$:

$$\frac{\partial \overline{g_i}}{\partial x} = \text{cov}\left(x, g_i\right) = \nu_i \tag{40}$$

and is the same value as we had for the Cramer-Rao derivation in Equation 28, when the gene expression g_i is Gaussian and conditional on the position x.

The log of the posterior distribution is given by:

$$\log p\left(x|g, g_n\right) = \log p\left(x\right) + \log p\left(g, g_n|x\right) + \text{const} \tag{41}$$

$$= -\frac{1}{2}\left[\frac{\left(x-\mu_x\right)^2}{\sigma_x^2} + \left(g - \mathbb{E}\left[g|x\right]\right)^T \left(\Sigma_g - \frac{1}{\sigma_x^2}\nu\nu^T\right)^{-1} \left(g - \mathbb{E}\left[g|x\right]\right)\right] + \text{const} \tag{42}$$

The second derivative of this log-posterior distribution with respect to x is equal to the negative inverse of the variance, and is given by:

$$\frac{1}{\sigma^2(x|g, g_n)} = -\frac{\partial^2 \log p\left(x|g_c, g_n\right)}{\partial x} = \frac{1}{\sigma_x^2} + \nu^T \left(\Sigma_g - \frac{1}{\sigma_x^2}\nu\nu^T\right)^{-1} \nu \tag{43}$$

The above expression can be further simplified using the matrix inversion lemma:

$$\left(\Sigma_g - \sigma_x^{-2}\nu\nu^T\right)^{-1} = \Sigma_g^{-1} + \frac{1}{\sigma_x^2}\frac{\Sigma_g^{-1}\nu\nu^T\Sigma_g^{-1}}{\sigma_x^2 + \nu\Sigma_g\nu^T} \tag{44}$$

Using this simplified form of the inverse of the matrix, the variance is equal to:

$$\sigma^2\left(\hat{x}|g, g_\ell\right) = \frac{1}{\frac{1}{\sigma_x^2} + \nu^T\Sigma_g^{-1}\nu + \frac{1}{\sigma_x^2}\nu^T\frac{\Sigma_g^{-1}\nu\nu^T\Sigma_g^{-1}}{\sigma_x^2+\nu\Sigma_g\nu^T}\nu} \tag{45}$$

$$= \frac{1}{\frac{1}{\sigma_x^2} + \nu^T\Sigma_g^{-1}\nu + \frac{1}{\sigma_x^2}\frac{\left(\nu^T\Sigma_g^{-1}\nu\right)^2}{\sigma_x^2+\nu\Sigma_g\nu^T}} \tag{46}$$

In our definition of the prior over the position x, it is centered around a specific point and approaches a uniform distribution when $\sigma_x^2 \to \infty$. Taking this limit, we get the following:

$$\lim_{\sigma_x^2\to\infty}\sigma^2\left(\hat{x}|g_c, g_n\right) = \frac{1}{\nu^T\Sigma_g^{-1}\nu} \tag{47}$$

This is the same as the lower bound in Section 3.

We can now use the fact that we have the explicit form of the bound in Equation 35. This allows to derive an exact expression for the information gap of the neighborhood-informed decoder under the Gaussian assumption:

$$I_{\text{gap}} = \log_2\left(\frac{N\sqrt{2\pi e}}{L}\cdot\sigma^2\left(\hat{x}|g, g_n\right)\right) \tag{48}$$

$$= \log_2\left(\frac{N\sqrt{2\pi e}}{L}\right) - \log_2\sigma^{-2}\left(\hat{x}|g, g_n\right) \tag{49}$$

$$= \log_2\left(\frac{N\sqrt{2\pi e}}{L}\right) - \log_2\left(\sigma^{-2}\left(\hat{x}|g\right) + \delta_{\text{neighbor}}\right) \tag{50}$$

5 Appendix figures

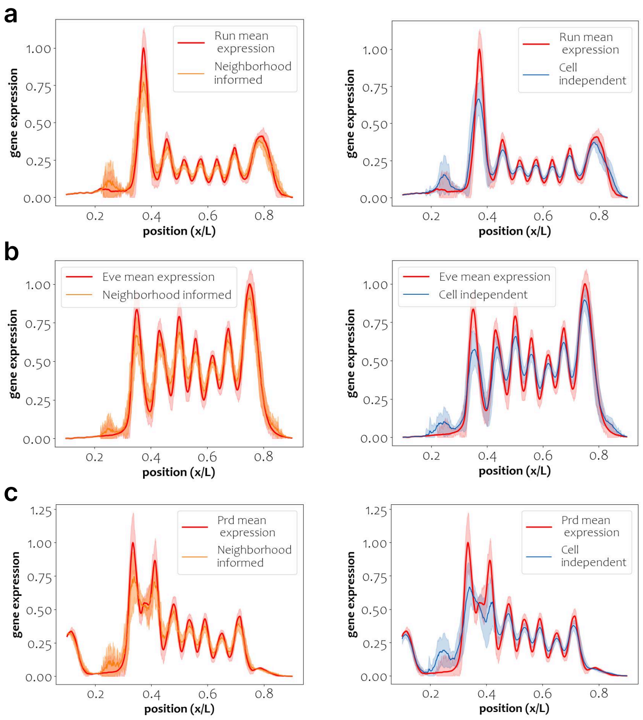

Appendix Figure S1: WT pair-rule gene expression predictions. The reconstruction of pair-rule gene expression spatial profiles based on the neighborhood-informed (orange) and cell-independent (blue) decoders, relative to the ground-truth mean pair-rule gene expression (red), for *(a)* Run, *(b)* Eve, and *(c)* Prd (also shown in Figure 3b).

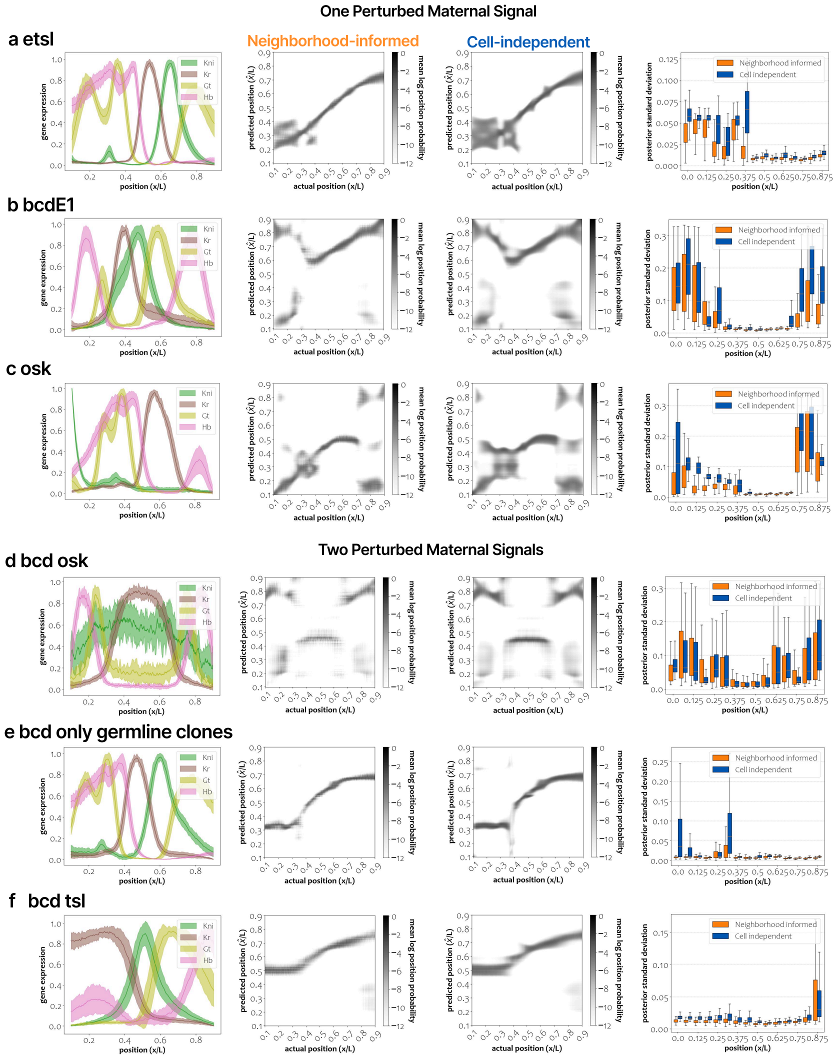

Appendix Figure S2: Position decoding and pair-rule stripe reconstruction in all mutant background embryos. Each row encases results for six types of mutant background embryos. The left most column shows gap gene expression profiles along the AP axis for each mutant type. The middle two columns show the decoding maps based on neighborhood-informed (left) and cell-independent (right) decoders. The right most column shows the posterior standard deviation over pair-rule expression reconstruction for the neighborhood-informed (orange) and cell-independent (blue) decoders, binned to 20 bins across the AP axis. Boxplots are defined between the 25% and 75% percentiles, with center line depicting the median and whiskers $\times 1.5$ the inter quartile range below and above the box. Results are shown for the following mutants: *(a)* etsl, *(b)* bcdE1, *(c)* osk, *(d)* bcd osk, *(e)* bcd only germline clones, and *(f)* bcd tsl. The posterior position distribution standard deviation is significantly reduced when decoding in a neighborhood-informed manner (t-test p-values between distribution of average standard deviations along the AP-axis: etsl $= 10^{-19}$, bcdE1 $= 6 \cdot 10^{-6}$, osk $= 10^{-86}$, bcd osk $= 5 \cdot 10^{-3}$, bcd only germlines $= 3 \cdot 10^{-15}$ and bcd tsl $= 2 \cdot 10^{-3}$). When comparing embryo-to-embryo, all mutants with a single perturbed maternal signal (etsl, bcdE1, and osk) had a statistically significant reduction in the standard deviation of the posterior distribution , as well as most instances of double perturbed maternal signal (100% of bcd only germline clones, 80% of bcd osk, and 40% of bcd tsl).

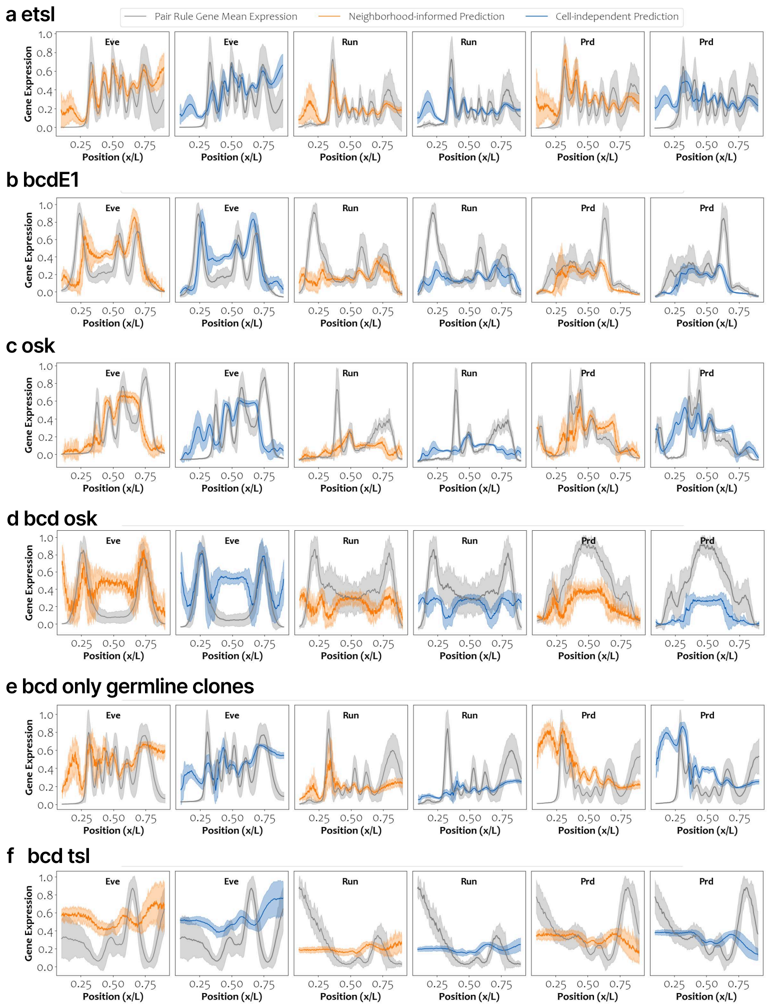

Appendix Figure S3: Pair-rule gene expression predictions in mutant background embryos. Each row presents the reconstruction of pair-rule gene expression spatial profiles based on the neighborhood-informed (orange) and cell-independent (blue) decoders, relative to the ground-truth mean pair-rule gene expression (gray), for Eve (first two columns), Run (next two columns), and Prd (last two columns). Each row corresponds to a different mutant type: *(a)* etsl, *(b)* bcdE1, *(c)* osk, *(d)* bcd osk, *(e)* bcd only germline clones, and *(f)* bcd tsl.

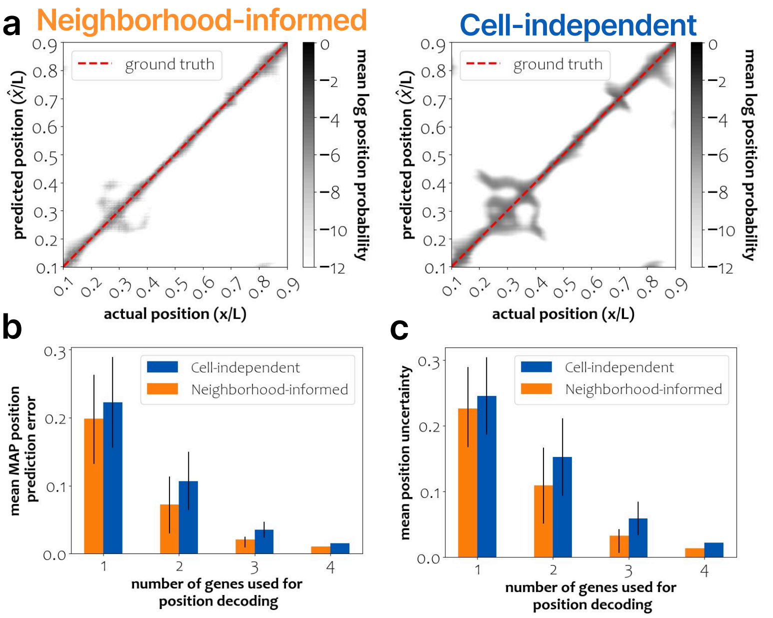

Appendix Figure S4: Position Decoding when training and testing on the same WT *Drosophila* embryo sample. The dataset of 38 WT embryos was used to infer the likelihood distribution and predict the positional posterior distribution. *(a)* Position decoding maps based on neighborhood-informed (left) and cell-independent (right) decoding given Kr, Gt, and Hb expression. *(b)* The mean error of position prediction using the MAP estimate over all $n_{\mathrm{WT}} = 38$ embryos, grouped by the number of gap genes used for decoding. Error-bars represent a single standard deviation over choice of genes used for position decoding. For each group, we compare neighborhood-informed to cell-independent decoding. The predicted error is significantly lower for neighborhood-informed decoding for all gene subsets. *(c)* The average standard deviation of the predicted position distribution over all $n_{\mathrm{WT}} = 38$, grouped by the number of gap genes used for decoding, is significantly lower for neighborhood-informed decoding for all gene subsets.

References

Dubuis JO, Samanta R, Gregor T (2013a) Accurate measurements of dynamics and reproducibility in small genetic networks. Molecular systems biology 9(1):639

Dubuis JO, Tkačik G, Wieschaus EF, et al (2013b) Positional information, in bits. Proceedings of the National Academy of Sciences 110(41):16301–16308

McGough L, Casademunt H, Nikolić M, et al (2024) Finding the last bits of positional information. PRX life 2(1):013016

Merle M, Friedman L, Chureau C, et al (2024) Precise and scalable self-organization in mammalian pseudo-embryos. Nature Structural & Molecular Biology pp 1–7

Petkova MD, Tkačik G, Bialek W, et al (2019) Optimal decoding of cellular identities in a genetic network. Cell 176(4):844–855

Tkačik G, Gregor T (2021) The many bits of positional information. Development 148(2):dev176065

Tkačik G, Dubuis JO, Petkova MD, et al (2015) Positional information, positional error, and readout precision in morphogenesis: a mathematical framework. Genetics 199(1):39–59

Wolpert L (1971) Positional information and pattern formation. Current topics in developmental biology 6:183–224

Zagorski M, Tabata Y, Brandenberg N, et al (2017) Decoding of position in the developing neural tube from antiparallel morphogen gradients. Science 356(6345):1379–1383
