# Supplementary material for: Neighborhood-informed positional information for precise cell identity specification
Source: Mol Syst Biol. 2026 May 5;22(7):1118–31. doi: 10.1038/s44320-026-00211-y (PMC13328375; doi:10.1038/s44320-026-00211-y)
Supplement: Supplementary file 3 — Source data Fig. 1 [file 44320_2026_211_MOESM3_ESM.zip › Figure 1/README.rtf]

All data was adapted from:Petkova, M. D., Tkačk, G., Bialek, W., Wieschaus, E. F., & Gregor, T. (2019). Optimal decoding of cellular identities in a genetic network. Cell, 176(4), 844-855.For data loading, normalization, and exact usage, see: https://github.com/nitzanlab/Neighborhood-Informed-Positional-Information
