# Supplementary material for: Neighborhood-informed positional information for precise cell identity specification
Source: Mol Syst Biol. 2026 May 5;22(7):1118–31. doi: 10.1038/s44320-026-00211-y (PMC13328375; doi:10.1038/s44320-026-00211-y)
Supplement: Supplementary file 7 — Source data Fig. 5 [file 44320_2026_211_MOESM7_ESM.zip › Figure 5/README.rtf]

Data for panels A-C can be found in:M. Merle, L. Friedman, C. Chureau, A. Shoushtarizadeh, and T. Gregor, Precise and scalable self-organization in mammalian pseudo-embryos, Nature Structural & Molecular Biology , 1 (2024).Data for panels D-E was received following communications with the authors of:M. Zagorski, Y. Tabata, N. Brandenberg, M. P. Lutolf, G. Tkaˇik, T. Bollenbach, J. Briscoe, and A. Kicheva, Decoding of position in the developing neural tube from antiparallel morphogen gradients, Science 356, 1379 (2017).For data loading, normalization, and exact usage, see: https://github.com/nitzanlab/Neighborhood-Informed-Positional-Information
