# Supplementary material for: Neighborhood-informed positional information for precise cell identity specification
Source: Mol Syst Biol. 2026 May 5;22(7):1118–31. doi: 10.1038/s44320-026-00211-y (PMC13328375; doi:10.1038/s44320-026-00211-y)
Supplement: Supplementary file 1 — Peer Review File [file 44320_2026_211_MOESM1_ESM.pdf]

molecular systems biology

Neighborhood-Informed Positional Information for Precise Cell Identity Specification

Michal Erez, Roy Friedman, and Mor Nitzan

Corresponding author(s): Mor Nitzan (mor.nitzan@mail.huji.ac.il)

Review Timeline:

Submission Date:	10th Feb 25
Editorial Decision:	2nd May 25
Revision Received:	14th Oct 25
Editorial Decision:	2nd Feb 26
Revision Received:	22nd Feb 26
Editorial Decision:	26th Feb 26
Revision Received:	15th Mar 26
Accepted:	15th Apr 26

Editor: Poonam Bheda

Transaction Report:

(Note: With the exception of the correction of typographical or spelling errors that could be a source of ambiguity, letters and reports are not edited. Depending on transfer agreements, referee reports obtained elsewhere may or may not be included in this compilation. Referee reports are anonymous unless the Referee chooses to sign their reports.)

2nd May 2025

Manuscript Number: MSB-2025-12910
Title: Neighborhood-Informed Positional Information for Precise Cell Identity Specification

Dear Dr Nitzan,

Thank you again for submitting your work to Molecular Systems Biology. We have now heard back from the two reviewers who agreed to evaluate your study. As you will see below, the reviewers appreciate that the proposed approach addresses a timely topic. However, they raise a series of concerns, which we would ask you to address in a major revision.

I think that the recommendations of the reviewers are rather clear and I therefore do not see the need to repeat the comments listed below. One of the more fundamental points raised by both reviewers refers to the need to extend the application to another system to test generalizability, preferably in another species. That being said, I discussed further in a cross-commenting session with the reviewers that experiments would not need to be performed for validation, and rather that analysis of publicly available datasets would be sufficient. All other issues raised would need to be satisfactorily addressed. Please let me know in case you would like to discuss in further detail any of the any of the reviewer comments or your proposed revisions, I would be happy to schedule a call.

When submitting your revised manuscript, please carefully review the instructions that follow below. We perform an initial quality control of all revised manuscripts before re-review; failure to include requested items will delay the evaluation of your revision.

We require:

1) A .docx formatted version of the manuscript text (including legends for main figures, EV figures and tables). Please make sure that the changes are highlighted to be clearly visible. Alternatively you may choose to submit your manuscript as a LaTeX file.

2) Individual production quality figure files as .eps, .tif, .jpg (one file per figure). For guidance, download the 'Figure Guide PDF' (https://www.embopress.org/page/journal/17574684/authorguide#figureformat).

3) At EMBO Press we ask authors to provide source data for the main figures. Our source data coordinator will contact you to discuss which figure panels we would need source data for and will also provide you with helpful tips on how to upload and organize the files.

4) A .docx formatted letter INCLUDING the reviewers' reports and your detailed point-by-point responses to their comments. As part of the EMBO Press transparent editorial process, the point-by-point response is part of the Peer Review File (PRF), which will be published alongside your paper.

5) A complete author checklist, which you can download from our author guidelines (https://www.embopress.org/page/journal/17574684/authorguide#submissionofrevisions). Please insert information in the checklist that is also reflected in the manuscript. The completed author checklist will also be part of the PRF.

6) Please note that all corresponding authors are required to supply an ORCID ID for their name upon submission of a revised manuscript.

7) It is mandatory to include a 'Data Availability' section after the Materials and Methods. Before submitting your revision, primary datasets produced in this study need to be deposited in an appropriate public database, and the accession numbers and database listed under 'Data Availability'. Please remember to provide a reviewer password if the datasets are not yet public (see https://www.embopress.org/page/journal/17574684/authorguide#dataavailability).

In case you have no data that requires deposition in a public database, please state so in this section as follows: "This study includes no data deposited in external repositories". Note that the Data Availability Section is restricted to new primary data that are part of this study.

8) All Materials and Methods need to be described in the main text using our 'Structured Methods' format, which is required for all research articles. According to this format, the Methods section includes a Reagents and Tools Table (listing key reagents, experimental models, software and relevant equipment and including their sources and relevant identifiers) followed by a Methods and Protocols section describing the methods using a step-by-step protocol format. The aim is to facilitate adoption of the methodologies across labs. Please upload the Reagents and Tools table as a separate document when submitting your revised manuscript. More information on how to adhere to this format as well as a downloadable template (.docx) for the Reagents and Tools Table can be found in our author guidelines:

https://www.embopress.org/page/journal/17444292/authorguide#structuredmethods

An example of a Method paper with Structured Methods can be found here:
https://www.embopress.org/doi/10.15252/msb.20178071.

9) For data quantification: please specify the name of the statistical test used to generate error bars and p-values, the number (n) of independent experiments (specify technical or biological replicates) underlying each data point and the test used to calculate p-values in each figure legend. The figure legends should contain a basic description of n, p-values and the test applied. Graphs must include a description of the bars and the error bars (s.d., s.e.m.). Please provide exact p-values (in either the figure or figure legend).

10) Our journal encourages inclusion of *data citations in the reference list* to directly cite datasets that were re-used and obtained from public databases. Data citations in the article text are distinct from normal bibliographical citations and should directly link to the database records from which the data can be accessed. In the main text, data citations are formatted as follows: "Data ref: Smith et al, 2001" or "Data ref: NCBI Sequence Read Archive PRJNA342805, 2017". In the Reference list, data citations must be labeled with "[DATASET]". A data reference must provide the database name, accession number/identifiers and a resolvable link to the landing page from which the data can be accessed at the end of the reference. Further instructions are available at .

11) We replaced Supplementary Information with Expanded View (EV) Figures and Tables that are collapsible/expandable online. EV Figures should be cited as 'Figure EV1, Figure EV2" etc... in the text and their respective legends should be included in the main text after the legends of regular figures.
- For the figures that you do NOT wish to display as Expanded View figures, they should be bundled together with their legends in a single PDF file called *Appendix*, which should start with a short Table of Content. Appendix figures should be referred to in the main text as: "Appendix Figure S1, Appendix Figure S2" etc.
- Additional Tables/Datasets should be labeled and referred to as Table EV1, Dataset EV1, etc. Legends should be provided in a separate tab in case of .xls files. Alternatively, the legend can be supplied as a separate text file (README) and zipped together with the Table/Dataset file.
See detailed instructions here:
https://www.embopress.org/page/journal/17574684/authorguide#expandedview

12) Author contributions: CRediT has replaced the traditional author contributions section because it offers a systematic machine-readable author contributions format that allows for more effective research assessment. Please remove the Authors Contributions from the manuscript and use the free text boxes beneath each contributing author's name in our system to add specific details on the author's contribution. More information is available in our guide to authors.

13) Disclosure statement and competing interests: We updated our journal's competing interests policy in January 2022 and request authors to consider both actual and perceived competing interests. Please review the policy https://www.embopress.org/competing-interests and update your competing interests if necessary.

14) Every published paper now includes a 'Synopsis' to further enhance discoverability. Synopses are displayed on the journal webpage and are freely accessible to all readers. They include a short stand first (maximum of 300 characters, including space) as well as 2-5 one-sentences bullet points that summarizes the paper. Please write the bullet points to summarize the key NEW findings. They should be designed to be complementary to the abstract - i.e. not repeat the same text. We encourage inclusion of key acronyms and quantitative information (maximum of 30 words / bullet point). Please use the passive voice. Please attach these in a separate file or send them by email, we will incorporate them accordingly.

Please also suggest a striking image or visual abstract to illustrate your article as a PNG file 550 px wide x 300-600 px high.

Please note that these would be the final versions and changes during proofing are usually not allowed.

15) As part of the EMBO Publications transparent editorial process initiative (see our policy here: https://www.embopress.org/transparent-process#Review_Process), Molecular Systems Biology will publish online a Peer Review File (PRF) to accompany accepted manuscripts.
In the event of acceptance, this file will be published in conjunction with your paper and will include the anonymous referee reports, your point-by-point response and all pertinent correspondence relating to the manuscript. Let us know whether you agree with the publication of the PRF and as here, if you want to remove or not any figures from it prior to publication.
Please note that the Author checklist will be published at the end of the PRF.

Molecular Systems Biology has a "scooping protection" policy, whereby similar findings that are published by others during review or revision are not a criterion for rejection. Should you decide to submit a revised version, I do ask that you get in touch after three months if you have not completed it, to update us on the status.

Yours sincerely,

Poonam Bheda, PhD
Scientific Editor
Molecular Systems Biology

Reviewer #1:

Summary & General remarks
The approach to estimate the contribution tissue neighborhoods to positional information is an interesting take that adds to a number of emerging fields in spatial omics. The manuscript is focussed on one application in drosophila embryogensis and explores spatial infromation beyond that of gap genes alone. This advance is well supported by a theoretical discussion of this neighborhood-derived positional information but lacks discussion on how well this can be estimated given the limitations of measurements of relevant systems. Nevertheless, we believe this work could be interesting for a wider audience at the interface of spatial omics and tissue patterning in embryogenesis if these application-centric details are improved.

Major points
1. After reading the manuscript, it is not clear enough what data was used to fit the models / perform the analyses and why this particular data set was chosen: e.g. "When analyzing the wild-type (WT) Drosophila embryo gap gene expression profiles measured in [8], we find that throughout the AP axis, the pairwise correlations in expression of both gap and pair-rule genes are indeed within the range needed to close the information gap (Equation 7; Figure 1b,c)." is the sole sentence on what data has ben used to arrive at this conclusion in the results section up until Fig. 1a-c, the reader needs to infer that this is likely a) a proteome readout, that is b) measured through microcospy, and c) processed based on cell segments or some binning along an axes through the embryo. Indeed, a lot of detail in the main text is spent on motivating theoretical ideas but too little on how these ideas may be limited by or are indeed possible to explore in this specific dataset - i.e. talk about data set size, variation across replicate embryos, observed gene expression variation along spatial strata (e.g. binned by "stripes"). These are not necessarily the analysis ideas that would be most suitable to motivate the specific analyses that the authors perform but similar exploratory analyses of the data complexity are likely beneficial as sanity checks. Note that we do not critique the choice of the experimental model, drosophila embryo, which does indeed seem like a great experimental model to study with this mathematical model, we are simply left wondering if this particular set of measurements of this system is particularly suitable for the analyses that have been performed.

2. On the point of identifiability of these statistical dependencies in this particular dataset - the manuscript lacks on a discussion of estimates of significance in key places where statements on the relevance on neighborhoods are made, for example here "While the positional decoding prediction error and the standard deviation of the position distribution decrease with increasing number of gap genes used for decoding for both decoder types (neighborhood-informed and cell-independent), the neighborhood-informed decoder consistently outperforms the independent decoder (Figure 1h,i)." A p-value is mentioned in in the caption, but this embryo-to-embryo comparison does not seem to reflect the (marginal?) data presented in the figure? This both needs to be consolidated and expanded upon in the main text as these comparisons to baseline models are effectively the key ablation performed in this study that justify the added complexity in accounting for the neighborhood, and thus distinguish this work from reference 8 as far as we understand.

3. The claims are based on on one setting - we will positively mention that this includes perturbations that are considered as a validation. However, given that this is a presentation of a mathematical model, we would like to see analyses such as a) a further application to a distinct measurement of drosophila embryogenesis to further corroborate that the claimed positional information is not an artefact of the measurements and/or b) an application to a experimental system other than Drosophila embryogenesis.

4. We would encourage the authors to comment on intrinsic determinants of gene expression states - the manuscript focusses on a setting in which the embryo is already partially patterened (i.e., gap gene expression is treated as a given background). In such a setting, one could arguably differentiate two hypotheses that lead to position-informative neighborhoods: a) that further positional patterns (beyond gap genes) were already imposed on cells and may reflect as particular molecular states of the cells, or b) that neighborhoods exert the remaining signal that is not yet given by gap genes. This sentence from the abstract "Here, we propose a neighborhood-informed information- theoretic framework where cells integrate local gene expression information as well as information from neighboring cells." suggests that the authors attribute the signal to the latter. Both would lead to correlations in the observational setting but may have different implications under perturbations and, importantly, reflect on whether the neighborhood-dependency that the authors estimate is a direct effect of this neighborhood or a direct effect of a latent variable that causes both the cell state and the neighborhood state.

5. There is a somewhat direct relationship between the neighborhood encoding and a encoding of neighborhoods in a spatial graph of cells that may be worth commenting on, as this may relate some of the information theoretic angles presented to graph

neural networks that are used in spatial transcriptomics.

Minor points
- Clarify wording of "prediction error" or "reconstruction error" on axes and in usage - is this MSE / RMSE?

Reviewer #2:

This manuscript introduces a neighborhood-informed positional information framework to explain how cells achieve precise identity specification during Drosophila embryogenesis. The study builds on prior work showing that gene expression patterns along the anterior-posterior (AP) axis encode positional information, but that individual cells alone do not have enough information to uniquely determine their positions. The authors propose that incorporating gene expression data from neighboring cells can help close this positional information gap, thereby improving the accuracy of position decoding.

Using an information-theoretic approach, the authors mathematically quantify the additional positional information available from neighboring gene expression patterns. Their analyses suggest that neighborhood-informed decoders predict cell positions with greater precision and lower variance than cell-independent decoders. They further test their framework on both wild-type and mutant Drosophila embryos, showing that neighborhood-informed decoding remains robust even in embryos with disrupted maternal signals. The study concludes that positional information is locally encoded within the embryo and that cells may use their microenvironment to refine their spatial identities.

As a reviewer with expertise in developmental biology rather than mathematical modeling, my comments focus on the biological interpretation of the findings rather than the technical aspects of the computational framework. Specifically, I am interested in how well the model aligns with known developmental mechanisms, whether the conclusions are sufficiently supported by experimental evidence, and how the findings contribute to our broader understanding of positional information in embryogenesis.

Major Comments
1. Lack of direct experimental validation. The study is primarily based on computational analyses, but I find that the biological relevance of the framework is not strongly supported by experimental data. While the authors use existing Drosophila gene expression datasets, they do not directly demonstrate that cells in vivo actually use neighboring information as predicted. It would be helpful if the authors provided experimental validation-such as live imaging, genetic perturbations, or single-cell lineage tracing-to confirm that cells integrate neighborhood information for positional identity specification. Without such evidence, the conclusions remain speculative, limiting the study's impact in developmental biology.

2. Uncertain biological relevance of positional accuracy improvements. While the study demonstrates that neighborhood-informed decoding improves positional accuracy, it is not clear whether this improvement has meaningful biological consequences. Positional information is critical for cell fate specification, but the manuscript does not directly test whether better decoding precision leads to more reliable fate determination or enhanced developmental robustness. It would be useful if the authors discussed how their findings relate to experimentally validated regulatory mechanisms or tested whether their predictions correlate with known developmental outcomes.

3. Overemphasis on mathematical derivations at the expense of biological interpretation.
I find that the manuscript focuses heavily on mathematical modeling and information theory, making it challenging for developmental biologists to interpret the biological significance of the results. While statistical improvements in positional accuracy are demonstrated, their functional relevance in embryonic development is not clearly explained. A more accessible discussion-linking the findings to known molecular mechanisms and comparing them to existing models of positional information-would help bridge the gap between theory and biology and make the study more approachable for a broader audience.

4. Need for sensitivity analysis to validate model assumptions. The theoretical framework relies on key assumptions, such as Gaussian gene expression distributions and Markovian spatial correlations, but it is unclear whether these assumptions hold in real embryos. Biological systems are inherently noisy, and if these statistical properties do not apply in vivo, the model's predictions may not be valid. A systematic sensitivity analysis, testing the robustness of the framework under different gene expression distributions and spatial correlation structures, would strengthen the study's conclusions.

5. Limited generalizability and lack of cross-species validation. The authors suggest that their framework could apply to other developmental systems, but they do not provide direct evidence beyond Drosophila. Many organisms use alternative mechanisms for cell fate specification, such as direct cell-cell signaling, mechanical forces, or spatially restricted regulatory networks, none of which are considered in this model. Given the increasing availability of spatial genomics datasets across various species, it would be beneficial for the authors to test their framework using publicly available data from other organisms. This would provide a broader assessment of whether neighborhood-informed positional decoding is a generalizable principle or specific to Drosophila.

Minor Comments
1. Improve accessibility for a biological audience. The manuscript is heavily mathematical, which may make it difficult for developmental biologists to follow. I suggest simplifying explanations where possible, providing intuitive biological interpretations, and clearly defining technical terms to improve readability for non-experts in mathematical modeling.

2. Refine citation approach. The manuscript frequently cites multiple references for a single point, sometimes making it difficult to identify the most relevant sources. The authors should prioritize citing original studies rather than secondary sources or review articles to improve clarity and avoid redundancy.

3. Expand the Discussion section. The Discussion is too brief and does not fully explore the broader implications of the findings. It would strengthen the manuscript if the authors expanded this section to discuss potential experimental validations, comparisons with other models of positional information, and possible applications beyond Drosophila.

4. Refine figure presentation. Some figures are overly dense, making it difficult to interpret the results clearly. It would be helpful if the authors split complex figures into multiple separate ones to improve readability and highlight key findings. There is ample opportunity to refine the layout for clarity.

Overall impression
This study presents an interesting framework for positional information decoding and introduces a novel perspective on how cells might integrate neighborhood information to refine spatial identities. However, the reliance on computational analyses without strong experimental validation makes it difficult to determine whether this framework reflects actual biological processes. Strengthening the empirical support, improving accessibility for biologists, and expanding the discussion on broader implications would significantly enhance the manuscript.

Dear Dr. Poonam Bheda,

We thank the reviewers for their overall positive evaluation, constructive comments, and suggestions. We have revised our manuscript accordingly, the main points of the revision are:

(1) We extended the analysis to two more developmental contexts beyond Drosophila: gastruloids derived from mouse embryonic stem cells and the developing neural tube (Figure 5).

(2) We added further discussion into biological consequences of our theoretical analysis and discussion related to the distinction of our work relative to previous studies.

(3) We simplified the description of the theoretical analysis in favor of more biological interpretations, where possible.

(4) We clarified and revised the text and figures, according to the reviewers' comments.

We have addressed all comments raised by the reviewers in the point-by-point response below, and have made the required revisions in the manuscript and supplementary material (marked in teal). We look forward to hearing from you.

Sincerely,
Michal, Roy, and Mor

Response to Reviewer #1:

Comment:
Summary & General remarks
The approach to estimate the contribution of tissue neighborhoods to positional information is an interesting take that adds to a number of emerging fields in spatial omics. The manuscript is focussed on one application in drosophila embryogenesis and explores spatial information beyond that of gap genes alone. This advance is well supported by a theoretical discussion of this neighborhood-derived positional information but lacks discussion on how well this can be estimated given the limitations of measurements of relevant systems. Nevertheless, we believe this work could be interesting for a wider audience at the interface of spatial omics and tissue patterning in embryogenesis if these application-centric details are improved.

Response: We thank the reviewer for their constructive comments. We have revised the manuscript accordingly, and provide detailed responses to all comments below.

Comment: After reading the manuscript, it is not clear enough what data was used to fit the models / perform the analyses and why this particular data set was chosen: e.g. "When analyzing the wild-type (WT) Drosophila embryo gap gene expression profiles measured in [8], we find that throughout the AP axis, the pairwise correlations in expression of both gap and pair-rule genes are indeed within the range needed to close the information gap (Equation 7; Figure 1b,c)." is the sole sentence on what data has been used to arrive at this conclusion in the results section up until Fig. 1a-c, the reader needs to infer that this is likely a) a proteome readout, that is b) measured through microscopy, and c) processed based on cell segments or some binning along an axes through the embryo. Indeed, a lot of detail in the main text is spent on motivating theoretical ideas but too little on how these ideas may be limited by or are indeed possible to explore in this specific dataset - i.e. talk about data set size, variation across replicate embryos, observed gene expression variation along spatial strata (e.g. binned by "stripes"). These are not necessarily the analysis ideas that would be most suitable to motivate the specific analyses that the authors perform but similar exploratory analyses of the data complexity are likely beneficial as sanity checks. Note that we do not critique the choice of the experimental model, drosophila embryo, which does indeed seem like a great experimental model to study with this mathematical model, we are simply left wondering if this particular set of measurements of this system is particularly suitable for the analyses that have been performed.

Response: To address this point and clarify the description of the data and the motivation for analyzing it, we clarified existing text and added text that better describes the data where relevant (for example, before the analysis in Subsection IIA and IIB). We have further moved a detailed description of the data from the Supplementary Information into the Methods, where dataset size and further characteristics are discussed in full.

Shortly, the wildtype data is comprised of quadruple antibody staining of N=254 Drosophila embryos for the trunk gap genes in the 38-48 minute time window, an additional N=38 embryos

in the narrower 40-44 minute window. Finally, another N=38 embryos were triple stained for the pair rule genes at the 45-55 minute time window. The expression levels were then extracted using image processing methods from immunofluorescence microscopy, after alignment of all embryos to the same axis.

We chose this data as it is particularly well suited to our analysis: the data contains numerous replicants (N=254 embryos), that were quadruple stained for all trunk gap genes, and imaged at high resolution. Moreover, the patterning of the Drosophila embryos mostly occurs across the AP axis, so that the signals of interest are 1-dimensional and easily aligned between individuals. These unique properties, together with the overall high quality of the data, enabled the fine-grained analysis we carried out in this work.

Finally, we added a description in the Discussion section which details the characteristics required of the measurements which can be analyzed through the framework described in the manuscript, as well as potential future extensions.

Comment: On the point of identifiability of these statistical dependencies in this particular dataset - the manuscript lacks on a discussion of estimates of significance in key places where statements on the relevance on neighborhoods are made, for example here "While the positional decoding prediction error and the standard deviation of the position distribution decrease with increasing number of gap genes used for decoding for both decoder types (neighborhood-informed and cell-independent), the neighborhood-informed decoder consistently outperforms the independent decoder (Figure 1h,i)." A p-value is mentioned in the caption, but this embryo-to-embryo comparison does not seem to reflect the (marginal?) data presented in the figure? This both needs to be consolidated and expanded upon in the main text as these comparisons to baseline models are effectively the key ablation performed in this study that justify the added complexity in accounting for the neighborhood, and thus distinguish this work from reference 8 as far as we understand.

Response: In the revised manuscript we describe the statistical tests and comparisons made in more details and report p-values wherever applicable. The relevant p-values we previously reported were for new Figure 2d/e (previously Figure 1h/i), Figure 3d and Figure 4e. In all of these, the statistical t-tests determined that the distribution of the results for the neighborhood-informed decoder is different from the cell-independent decoder.

For Figure 2d and e, the statistical tests do not relate to the error bars in the figure itself (which represent the mean and standard deviation over all choices of the possible genes), but rather to the inter-embryo deviations. In Figure 2d, the error in the predicted position is compared, while in Figure 2e the positional error is considered. This distinction is now clarified as part of the main text and also in the figure caption. For all 15 possible subsets of genes (of each possible size), the neighborhood-informed decoder performs significantly better than the cell-independent decoder under a t-test, with a p-value smaller than 0.05.

For Figures 3d and 4e, the statistical test, a standard t-test, is between the errors accrued when reconstructing the respective pair-rule genes using the decoder's estimate of the position, for WT embryos in Figure 3d, and mutant embryos in Figure 4e. As above, in both cases, for the pair-rule genes considered, the difference between the two distributions are statistically significant, with a p-value smaller than 0.05.

Comment: The claims are based on one setting - we will positively mention that this includes perturbations that are considered as a validation. However, given that this is a presentation of a mathematical model, we would like to see analyses such as a) a further application to a distinct measurement of drosophila embryogenesis to further corroborate that the claimed positional information is not an artefact of the measurements and/or b) an application to a experimental system other than Drosophila embryogenesis.

Response: The reviewer raises an important point, and we also believe that the addition of further biological contexts would greatly strengthen our claims and have strived to do exactly that. In the revised manuscript, we have added analyses of the positional information for two additional systems, gastruloids (new Figure 5a-c), with data from [1], and the developing neural tube (new Figure 5d-f) from [2]. The results for these new systems appear in a new Results section in the main text, Figure 5, and a detailed description of this new data can be found in the Methods section. In both of these systems, we again show that the positional information implied by the neighborhood-informed decoder is significantly higher (both with a p-value < 0.001 under a standard t-test) than that of previously explored (cell-independent) notions of positional information. Along most of the patterning axis for both systems, the neighborhood-informed decoder enables unique cell specification whereas the cell-independent decoder does not.

In the gastruloids data, we found that the cell-independent decoder contains around $I_{CI} = 4.6 \pm 1.1$ bits of positional information and the neighborhood-informed decoder $I_{NI} = 6.8 \pm 0.6$, while the amount of information required for unique cell-specification is $I_{unique} = 5.2 \pm 0.2$. Thus, the neighborhood-informed decoder is able to uniquely specify cell locations along the whole AP axis, whereas the cell-independent decoder is not.

For the developing neural tube, we found that the cell-independent decoder explains $I_{CI} = 4.5 \pm 0.9$ bits of information out of the $I_{unique} = 5.4 \pm 0.1$ bits needed for unique cell specification. On the other hand, the neighborhood-informed decoder explains $I_{NI} = 6.1 \pm 0.8$ bits, larger on average than the amount required for unique specification of positions.

We believe that this new addition strengthens our overall approach and results, a full discussion of this is in the revised Methods section.

Comment: We would encourage the authors to comment on intrinsic determinants of gene expression states - the manuscript focuses on a setting in which the embryo is already partially patterned (i.e., gap gene expression is treated as a given background). In such a setting, one could arguably differentiate two hypotheses that lead to position-informative neighborhoods: a)

that further positional patterns (beyond gap genes) were already imposed on cells and may reflect as particular molecular states of the cells, or b) that neighborhoods exert the remaining signal that is not yet given by gap genes. This sentence from the abstract "Here, we propose a neighborhood-informed information- theoretic framework where cells integrate local gene expression information as well as information from neighboring cells." suggests that the authors attribute the signal to the latter. Both would lead to correlations in the observational setting but may have different implications under perturbations and, importantly, reflect on whether the neighborhood-dependency that the authors estimate is a direct effect of this neighborhood or a direct effect of a latent variable that causes both the cell state and the neighborhood state.

Response:
We thank the reviewer for this comment - we agree that this is an important observation/ clarification, and we now relate to it in the manuscript. Specifically, first - when we relate in the manuscript to a ‘cell-independent’ or ‘neighborhood-informed’ decoders, the input to both decoders are only gap gene expression levels, either of individual cells or of the cells and their neighbors. This assumption is based on a series of previous works (e.g. [3,4,5]) which have used gap gene expression input to reason about positional decoding in embryos. We have clarified this and added this point to the discussion section, stating that in alternative scenarios, different systems, or given different experimental data, additional types of inputs could potentially affect cellular state (including in a cell-independent setting), and could be taken into account. Furthermore, we would also like to note that our framework is not specific to a particular information transfer / interaction mechanism. Given this, we agree that in general, either scenario is possible - spatial patterns were already inherent to the cells at the time the data was collected; or the neighborhood enables cells to be further localized, which helps the development of patterns. Further perturbation experiments in future work could help distinguish the two possibilities, as we now mention in the revised Discussion. We would like to clarify that in this study our main aim was to show that positional information exists in local neighborhoods, and not to claim that it is the only possible mechanism which can give rise to the highly precise spatial patterning empirically observed. We now additionally add this this point and clarification to the Discussion.

Comment: There is a somewhat direct relationship between the neighborhood encoding and a encoding of neighborhoods in a spatial graph of cells that may be worth commenting on, as this may relate some of the information theoretic angles presented to graph neural networks that are used in spatial transcriptomics.

Response: Thank you for the suggestion. There is indeed a connection between our analysis and graph-based methods in spatial data. While our theoretical analysis focused on 1D chains for simplicity, we believe it can be generalized to more complex graph structures. We have added a short exploration of this idea in the discussion.

Comment: Clarify wording of "prediction error" or "reconstruction error" on axes and in usage - is this MSE / RMSE?

Response:
The "prediction error" referred to in the text is the average error of the position predicted by the decoder across the AP axis, where the error is the absolute value of the difference between the true and predicted locations. Similarly, the "reconstruction error" is the average error of the reconstructed pair-rule expression across the AP axis, where the error is again the absolute value between the true and predicted values. We have revised the manuscript to clarify this point in the main text, as well as in the Methods section.

Response to Reviewer #2:

Comment:
This manuscript introduces a neighborhood-informed positional information framework to explain how cells achieve precise identity specification during Drosophila embryogenesis. The study builds on prior work showing that gene expression patterns along the anterior-posterior (AP) axis encode positional information, but that individual cells alone do not have enough information to uniquely determine their positions. The authors propose that incorporating gene expression data from neighboring cells can help close this positional information gap, thereby improving the accuracy of position decoding.

Using an information-theoretic approach, the authors mathematically quantify the additional positional information available from neighboring gene expression patterns. Their analyses suggest that neighborhood-informed decoders predict cell positions with greater precision and lower variance than cell-independent decoders. They further test their framework on both wild-type and mutant Drosophila embryos, showing that neighborhood-informed decoding remains robust even in embryos with disrupted maternal signals. The study concludes that positional information is locally encoded within the embryo and that cells may use their microenvironment to refine their spatial identities.

As a reviewer with expertise in developmental biology rather than mathematical modeling, my comments focus on the biological interpretation of the findings rather than the technical aspects of the computational framework. Specifically, I am interested in how well the model aligns with known developmental mechanisms, whether the conclusions are sufficiently supported by experimental evidence, and how the findings contribute to our broader understanding of positional information in embryogenesis.

Response: We thank the reviewer for their comments and greatly appreciate their biologically-focused suggestions and input.

Comment: Lack of direct experimental validation. The study is primarily based on computational analyses, but I find that the biological relevance of the framework is not strongly supported by experimental data. While the authors use existing Drosophila gene expression datasets, they do not directly demonstrate that cells in vivo actually use neighboring information as predicted. It would be helpful if the authors provided experimental validation-such as live imaging, genetic perturbations, or single-cell lineage tracing-to confirm that cells integrate neighborhood information for positional identity specification. Without such evidence, the conclusions remain speculative, limiting the study's impact in developmental biology.

Response: We agree with the reviewer that direct experimental validation would be a strong further step in our analysis, but believe it is out of scope for this current work. We have fleshed out the Discussion section with a more in-depth discussion as to the biological relevance of our work. Briefly, we now contextualize our work, referring to prior works on cell interactions during development, either through direct cell-cell interactions [6], or indirect signaling mechanisms, such as the alteration of adhesive [7] or mechanical properties (e.g. elasticity) of the cells [8], which can affect cell fate decisions during development, as we analyze in the current work through the analysis of positional information using information-theoretic tools. Our analysis, which is agnostic to the particular type of interaction mechanism, showed that enough information to unique specify cellular location exists within local microenvironments.

Beyond the previous analysis of development in Drosophila embryos, we have added two new biological contexts, gastruloids [1] and the developing neural tube [2] (Section 2F, Figure 5). We illustrate how our analysis can be carried over to those contexts, where we found similar results as the ones we established for the Drosophila embryo. In particular, local neighborhoods in both gastruloids and the developing neural tube contain additional information regarding the position of single cells or nuclei, in most scenarios to the extent that it is possible to uniquely specify the position of each cell (for gastruloids, the neighborhood-informed decoder has $I_{NI} = 6.8 \pm 0.6$ bits of information and the cell-independent has $I_{CI} = 4.6 \pm 1.1$ when $I_{unique} = 5.2 \pm 0.2$ are enough to uniquely specify location; for the neural tube, these are $I_{NI} = 6.1 \pm 0.7$, $I_{CI} = 4.5 \pm 0.9$ and $I_{unique} = 5.4 \pm 0.1$ respectively). Please see our response to the comments below (and the revised manuscript) for more details on these new systems.

We want to emphasize that our main focus was to show that the positional information needed to uniquely identify cell position exists in local neighborhoods, and do not attempt to infer which particular mechanism is employed by cells to incorporate this information. We view this as an advantage of our study - regardless of the specific mechanism of sharing information, we were still able to illustrate that such information can help cells better localize in space. This is a direct continuation of previous work [4] which showed that such information is missing in the standard analysis of positional information, despite ample empirical evidence that cells have the effective ability to estimate their position uniquely along the patterning axis [5]. Our aim was to show that this information might be embedded in the local environment of each cell, and to show that such information is indeed enough to uniquely specify cell locations.

In the revised manuscript, we have extended the Discussion section to better indicate the above.

Comment: Uncertain biological relevance of positional accuracy improvements. While the study demonstrates that neighborhood-informed decoding improves positional accuracy, it is not clear whether this improvement has meaningful biological consequences. Positional information is critical for cell fate specification, but the manuscript does not directly test whether better decoding precision leads to more reliable fate determination or enhanced developmental robustness. It would be useful if the authors discussed how their findings relate to experimentally validated regulatory mechanisms or tested whether their predictions correlate with known developmental outcomes.

Response: In various developmental contexts, and for Drosophila embryos in particular, the patterning of gene expression levels is highly reproducible across individuals. The precision of this patterning is to the level that variance of stripe position between different individuals is approximately half the internuclear distance [9]. As described in [4], this implies that each cell has enough information to uniquely determine its position, which does not align with previous notions and results of cell-independent positional information. Our analysis into the amount of positional information, which is invariably related to the precision of the positional decoder, attempts to explain exactly this phenomenon. As such, we should expect that the decoder is precise enough in order to uniquely specify cell location. In the revised manuscript, we added a detailed description of the above to the introduction, and as part of the discussion.

Comment: Overemphasis on mathematical derivations at the expense of biological interpretation.
I find that the manuscript focuses heavily on mathematical modeling and information theory, making it challenging for developmental biologists to interpret the biological significance of the results. While statistical improvements in positional accuracy are demonstrated, their functional relevance in embryonic development is not clearly explained. A more accessible discussion-linking the findings to known molecular mechanisms and comparing them to existing models of positional information-would help bridge the gap between theory and biology and make the study more approachable for a broader audience.

Response: We thank the reviewer for the suggestion. In the revised manuscript, we have removed the reliance on mathematical expressions, where possible, in favor of biological intuition. Additionally, we have expanded the introduction to positional information to better explain what was missing in previous theories of positional information, in the first subsection of the Results section. Finally, we have expanded the Discussion section to better frame our work in the context of existing work, link the findings to potential interaction mechanisms, comparing neighborhood-informed positional information more intuitively to existing models of positional information.

Comment: Need for sensitivity analysis to validate model assumptions. The theoretical framework relies on key assumptions, such as Gaussian gene expression distributions and Markovian spatial correlations, but it is unclear whether these assumptions hold in real embryos. Biological systems are inherently noisy, and if these statistical properties do not apply in vivo, the model's predictions may not be valid. A systematic sensitivity analysis, testing the robustness of the framework under different gene expression distributions and spatial correlation structures, would strengthen the study's conclusions.

Response: In the revised manuscript, we have added a clearer description of the Gaussian and Markovian assumptions and their biological motivation. Of these, previous works [3] showed that for each location, the distribution of gene expression is approximately Gaussian. Additionally, it was also shown that the noise in the positional decoding is also approximately Gaussian [4]. We now relate to these points in the revised manuscript.

Comment: Limited generalizability and lack of cross-species validation. The authors suggest that their framework could apply to other developmental systems, but they do not provide direct evidence beyond Drosophila. Many organisms use alternative mechanisms for cell fate specification, such as direct cell-cell signaling, mechanical forces, or spatially restricted regulatory networks, none of which are considered in this model. Given the increasing availability of spatial genomics datasets across various species, it would be beneficial for the authors to test their framework using publicly available data from other organisms. This would provide a broader assessment of whether neighborhood-informed positional decoding is a generalizable principle or specific to Drosophila.

Response: We agree that adding examples beyond Drosophila embryos could indeed strengthen the paper. To this end, in the revised manuscript we consider two additional developmental contexts for the analysis of neighborhood-informed positional information - , gastruloids (new Figure 5a-c), with data from [1], and the developing neural tube (new Figure 5d-f) from the study [2]. Briefly, in both systems, we again found that incorporating information from local neighborhoods enables better localization in space for each cell. In both systems, the neighborhood-informed decoder enables unique cell specification over most of the patterning axis (AP for gastruloids and dorsal-ventral for the neural tube) whereas the cell-independent decoder does not.

Quantitatively, in the gastruloids system we found that the cell-independent decoder contains around $I_{CI} = 4.6 \pm 1.1$ bits of positional information and the neighborhood-informed decoder $I_{NI} = 6.8 \pm 0.6$, while the amount of information required for unique cell-specification is $I_{unique} = 5.2 \pm 0.2$. Thus, the neighborhood-informed decoder is able to uniquely specify cell locations along the whole AP axis, whereas the cell-independent decoder is not. Similarly, for the developing neural tube, the cell-independent decoder explains $I_{CI} = 4.5 \pm 0.9$ bits of information out of the $I_{unique} = 5.4 \pm 0.1$ bits needed for unique cell specification. On the other hand, the neighborhood-informed decoder explains $I_{NI} = 6.1 \pm 0.7$ bits, above the amount required for unique specification of positions.

These experiments have been added as a new subsection in the Results (subsection F). At the same time, we have added a full description of these datasets to the Methods.

Comment: Improve accessibility for a biological audience. The manuscript is heavily mathematical, which may make it difficult for developmental biologists to follow. I suggest simplifying explanations where possible, providing intuitive biological interpretations, and clearly defining technical terms to improve readability for non-experts in mathematical modeling.

Response: We thank the reviewer for this important point. In the revised manuscript, we aimed to give better biological intuitions and discussions in each of the relevant sections. Additionally, where possible, we simplified the math and only introduced mathematical concepts that are essential to the understanding of our results (this led, for example, to the substantial shortening and clarification of the first Results section, where we moved some of the mathematical details to the Appendix).

Comment: Refine citation approach. The manuscript frequently cites multiple references for a single point, sometimes making it difficult to identify the most relevant sources. The authors should prioritize citing original studies rather than secondary sources or review articles to improve clarity and avoid redundancy.

Response: Thank you for the suggestion. In the revised manuscript, we strived to make more pointed citations, where possible.

Comment: Expand the Discussion section. The Discussion is too brief and does not fully explore the broader implications of the findings. It would strengthen the manuscript if the authors expanded this section to discuss potential experimental validations, comparisons with other models of positional information, and possible applications beyond Drosophila.

Response: As mentioned above, we have fleshed out the revised Discussion section to more fully explore the biological implications of our work, discuss applications beyond Drosophila (which we have also added to the Results section), shortly discuss potential experimental validations, expand the discussion about the data and its limitations, and finally we better frame the novelty with respect to previous work in the field.

Comment: Refine figure presentation. Some figures are overly dense, making it difficult to interpret the results clearly. It would be helpful if the authors split complex figures into multiple separate ones to improve readability and highlight key findings. There is ample opportunity to refine the layout for clarity.

Response: We thank the reviewer for the suggestion, and following it we have simplified and refined the presentation and description of figures in the revised manuscript, including adjusting captions and splitting Figure 1 of the previous version into two, Figures 1 and 2 in the revised manuscript.

References:

1. Merle, Melody, et al. "Precise and scalable self-organization in mammalian pseudo-embryos." Nature Structural & Molecular Biology 31.6 (2024): 896-902.
2. Zagorski, Marcin, et al. "Decoding of position in the developing neural tube from antiparallel morphogen gradients." Science 356.6345 (2017): 1379-1383.
3. Dubuis, Julien O., et al. "Positional information, in bits." Proceedings of the National Academy of Sciences 110.41 (2013): 16301-16308.
4. McGough, Lauren, et al. "Finding the last bits of positional information." PRX life 2.1 (2024): 013016.
5. Petkova, Mariela D., et al. "Optimal decoding of cellular identities in a genetic network." Cell 176.4 (2019): 844-855.
6. Su, Jimeng, et al. "Cell–cell communication: new insights and clinical implications." Signal transduction and targeted therapy 9.1 (2024): 196.
7. Tsai, Tony Y-C., et al. "An adhesion code ensures robust pattern formation during tissue morphogenesis." Science 370.6512 (2020): 113-116.
8. Heisenberg, Carl-Philipp, and Yohanns Bellaïche. "Forces in tissue morphogenesis and patterning." Cell 153.5 (2013): 948-962.
9. Dubuis, Julien O., Reba Samanta, and Thomas Gregor. "Accurate measurements of dynamics and reproducibility in small genetic networks." Molecular systems biology 9.1 (2013): 639.

2nd Feb 2026

Manuscript Number: MSB-2025-12910R
Title: Neighborhood-Informed Positional Information for Precise Cell Identity Specification

Dear Dr Nitzan,

Thank you again for submitting your revised work to Molecular Systems Biology. We have now heard back from the one of the original two reviewers who evaluated your study, Reviewer 2, as well as a new reviewer, Reviewer 3. Both Reviewer 2 and Reviewer 3 were asked to look over Reviewer 1's comments as well as the responses you provided in your point-by-point. While while usually new comments should not be raised at a Revision stage, we do not overrule if there are potential technical concerns; therefore, we reached out to Reviewer 2 again to arbitrate and provide guidance as to whether the concerns raised by Reviewer 3 were indeed technical or conceptual. From their point of view, they felt that Reviewer 3 had a difference in disciplinary perspective having to do with framing, terminology, and methodological interpretation, rather than pointing to a clear mathematical or implementation-level problem. However, they also felt that Reviewer 3 raised a fair and important point that some formulations in the current manuscript could be read as implying mechanistic cell-cell communication, which is not something the analysis directly demonstrates and would benefit from more explicit clarification or adjustment. They felt similarly regarding the comments on probabilistic decoding versus deterministic developmental programs and suggested that a clearer articulation on why a probabilistic framework is appropriate in this context would strengthen the paper, along with where its biological interpretive limits lie.

Given the overall comments by Reviewer 3 and the evaluation on the validity of the comments by Reviewer 2, we would ask you to revise the manuscript beyond simple cosmetic editing to tighten key claims, more carefully scope the conclusions, and position the work more explicitly as a computational contribution with limited mechanistic inference, with the guidance of Reviewer 2's points below.

Please let me know in case you would like to discuss in further detail any of the any of the reviewer comments, I would be happy to schedule a call. When submitting your revised manuscript, please provide a point-by-point to all of the reviewer comments (all Reviewer 2 and Reviewer 3 comments).

Yours sincerely,

Poonam Bheda, PhD
Scientific Editor
Molecular Systems Biology

Reviewer #2:

The authors have provided a clear and thoughtful point-by-point response. I appreciate their substantial effort made to address the reviewers' concerns.
In particular, I would like to highlight the following positive aspects:

Extension to additional systems: The incorporation of two additional developmental contexts (mouse gastruloids and the neural tube) broadens the applicability of the model and reinforces its biological relevance beyond the original Drosophila framework.

Improved accessibility: The authors have taken meaningful steps to improve clarity, simplifying the mathematical exposition where appropriate and offering more intuitive biological interpretations. These changes make the manuscript more approachable for a broader developmental biology readership.

Clarification and refinement: The text and figures have been revised for clarity, and the overall structure is now more coherent and easier to follow.

Overall, I find that the manuscript has been significantly improved and now presents the work in a more balanced and accessible manner.

Reviewer #3:

The manuscript MSB-2025-12910R addresses one of the most hot and interesting problems in developmental biology- the question of coding and decoding of positional information. The presented work bases mostly on two papers : one from J. O. Dubuis et al., 2013, and another from L. McGough et al., 2024.
The main points of these papers are that positional information is encoded in gene expression patterns (with example of gap-genes) and that Drosophila embryos can utilize this information to decode their position along the anterior-posterior axis with 1% accuracy.
In the presented MS the authors claim that this precision is insufficient to uniquely determine cell position in embryo, which leads to « a positional information gap ». In order to discover the additional information which should account for such a « gap », they propose « a neighborhood-informed information-theoretic framework where cells integrate local gene expression information as well as information from neighboring cells ».
I absolutely agree, that this problem exists and that it is extremely interesting and important. But I think that the presented work does not provide an answer to the question posed.
The major discrepancy in the story is that in order to introduce additional « layer » of positional information the authors proposed adding the information from neighboring cells. They even call it « cell-cell interaction ». But in fact what they have really added to the work of L. McGough et al., 2024, reported that positional information of AP axis depends on expression of gap-genes , is the information of expression of pair-rule genes, being calculated in cells across the AP axis.
But gene expression patterns in the neighboring cells is not the information which can be read or received by one cell from another ; this is not « neighboring cells information » neither a « cell-cell interaction » information. Thus, all mathematical formulas used for describing this wrong basic conception seem to be useless.
The idea that « information for cells to specify their position exists in their local neighborhood » is well known and is developing a lot in biology. Introducing an additional layer of positional information based on this idea and its mathematical formalization is a very breakthrough task, but here we see only a using of a well known information about patterns of expression of 2 families of genes in AP axis of Drosophila embryo.
Moreover, the mathematical part itself also has its weaknesses, which is not necessary to describe having the major problem mentioned above. Just to start with the question to the authors : why « the amount of positional information encoded in local expression levels canbe inferred from the performance of the optimal probabilistic position decoder given the gene expression in a certain position and its microenvironment » ? Why probabilistic ? The main challenge of positional information" problem lies in its deterministic nature. The gene expression network is organized to provide specific structures; it is not a probabilistic process requiring optimization.
Also, one of the clear weaknesses of the manuscript: it lacks a necessary summary of the main points from that 2 papers, on which the entire story is based (one from PNAS, 2013, and another from L. McGough et al., 2024), giving just a references to them.Thus the manuscript can not be read and understood as an entire independent report: in order to read and understand all the details and to form an adequate opinion, it turns out that it is needed to find and read these two papers. The same is true for the part of the MS containing mice data.
Among other minuses (which could be crucial even without fundumental drawbacks above)there are :
- applying the analysis only to AP axis, at the short time period of Drosophilla embryo development (which is not clearly specified in the text) ; for Drosophilla DV axis this analysis is not applicable ? for Drosophilla AP axis at other time periods this analysis is not applicable ? Why do you jump to mice?
- all story about mice embryos seems to be superfluous ; it is not well described, and not clear why two different objects were used.
- it is a striking difference between « gene expression patterns » in individual cells and « signaling gradients of proteins » (such as BMP and Shh) accross an embryo.
- the most minor issue: having the term « gap » for both « gap-genes » and « information gap » seems to be not a sophisticated choice

Reviewer 2 comments on author response to Reviewer 1:
Overall, the reviewer is generally positive about the conceptual approach and considers the framework relevant to current work in spatial omics and embryonic patterning. The comments do not challenge the core idea or the validity of the model, but instead focus on the clarity of data usage, statistical interpretation, and the scope of applicability. Most points are raised as requests for clarification or additional context rather than as fundamental objections. The reviewer indicates that the manuscript could be of interest to a broader audience if these application-related aspects are sufficiently clarified.

Assessment of the responses to Reviewer #1's comments:
Comment 1: The authors have improved the description of the dataset and clarified how the data were obtained and processed. However, the extent to which the specific measurements quantitatively support the analyses is only partially addressed, as no additional exploratory or sanity-check analyses of data variability are included.
Comment 2: The statistical testing and the relationship between the reported p-values and the figures are clarified. The response focuses mainly on technical details of the tests and does not fully address the reviewer's point that these comparisons represent the primary ablation distinguishing this work from prior models.
Comment 3: The concern regarding generality is addressed by extending the analysis to two additional developmental systems,

providing independent support that the reported effects are not limited to a single dataset or organism.
Comment 4: The authors acknowledge the potential ambiguity between neighborhood effects and latent intrinsic determinants and limit the scope of their claims accordingly. Some abstract-level phrasing may still be interpreted as implying a causal relationship, depending on wording.
Comment 5: The conceptual connection to graph-based spatial models is acknowledged and briefly discussed. Given the exploratory nature of the comment, this response appears sufficient.
Comment 6: The definitions of prediction and reconstruction errors are clarified in the main text and Methods, resolving the ambiguity raised by the reviewer.

Overall, the authors have responded constructively to the reviewer's comments and have addressed most points at an appropriate level. Several concerns are fully resolved through clarification of terminology, statistical testing, and the addition of analyses in additional biological systems. A small number of points remain only partially addressed and relate mainly to framing and interpretation rather than to missing analyses.
To further strengthen the revision without requesting new data, it may be helpful to ask the authors to (i) briefly note the limitations of the current measurements when motivating the quantitative analyses, (ii) state more explicitly in the main text that the comparison between neighborhood-informed and cell-independent decoders represents the main ablation underlying the framework, and (iii) review abstract-level wording to avoid or clearly qualify causal interpretations of neighborhood effects. These points could likely be addressed through targeted textual revisions in the Abstract, Results, and Discussion.

Reviewer 2 comments (partially quoted and partially paraphrased) on comments from Reviewer 3:

Please address Reviewer 3's conceptual concerns explicitly through targeted revision (rather than by introducing additional analyses), with the following points as guidance:

(1) Conceptual framing and terminology. It would be helpful to revisit language that could be read as implying direct cell-cell interaction or molecular readout, and to state more explicitly that "neighborhood information" is meant to refer to statistical correlations within an information-theoretic decoding framework. The authors could also identify places where the statistical availability of information might be interpreted as biological accessibility at the single-cell level, and to clarify or temper these statements accordingly.

(2) Methodological scope and interpretation. Please provide a clearer justification of the probabilistic decoding approach, including a brief discussion of why this framework is appropriate for the biological question being addressed, as well as an explicit acknowledgment of its limitations. It would also be useful for the authors to more clearly distinguish which conclusions are directly supported by the computational analysis and which should be presented as hypothesis-generating rather than mechanistically established.

(3) Contextualization of prior work. The manuscript would benefit from a concise but explicit summary of the core findings of Dubuis et al. (2013) and McGough et al. (2024), so that it can be read as a more self-contained contribution. In addition, please clarify more precisely how their framework extends, reinterprets, or differs from these prior studies.

(4) Biological scope and narrative limits. It may be important for the authors to clearly specify the developmental stages, spatial domains, and axes to which their analysis applies, and to avoid extrapolating beyond those boundaries. With regard to the mouse data, the authors could either strengthen the conceptual rationale for its inclusion or, alternatively, present it more explicitly as a limited illustrative extension rather than a central validation of the framework.

(5) Terminology and presentation. It would be beneficial to revise potentially confusing or overloaded terminology (for example, the use of "gap" in multiple contexts), which would help reduce conceptual ambiguity and improve clarity for a broad readership.

Dear Dr. Poonam Bheda,

We again thank all reviewers for reviewing our manuscript and providing constructive feedback.

We thank Reviewer 2 for their positive evaluation and suggestions, as well as their helpful guidance regarding the comments of both Reviewer 1 and Reviewer 3. We have addressed all of these comments in the point-by-point response below, and have made the required revisions in the manuscript (marked in teal). We look forward to hearing from you.

Sincerely,
Michal, Roy, and Mor

Reviewer 2 comments:

The authors have provided a clear and thoughtful point-by-point response. I appreciate their substantial effort made to address the reviewers' concerns.

In particular, I would like to highlight the following positive aspects:

Extension to additional systems: The incorporation of two additional developmental contexts (mouse gastruloids and the neural tube) broadens the applicability of the model and reinforces its biological relevance beyond the original Drosophila framework.

Improved accessibility: The authors have taken meaningful steps to improve clarity, simplifying the mathematical exposition where appropriate and offering more intuitive biological interpretations. These changes make the manuscript more approachable for a broader developmental biology readership.

Clarification and refinement: The text and figures have been revised for clarity, and the overall structure is now more coherent and easier to follow.

Overall, I find that the manuscript has been significantly improved and now presents the work in a more balanced and accessible manner.

Response: We thank the reviewer for their appreciation of our manuscript, their constructive suggestions over the course of the review process, and we are happy to hear that they found the new version of the manuscript clearer, with broadened applicability, and significantly improved.

Reviewer 2 comments on author response to Reviewer 1:
Overall, the reviewer is generally positive about the conceptual approach and considers the framework relevant to current work in spatial omics and embryonic patterning. The comments

do not challenge the core idea or the validity of the model, but instead focus on the clarity of data usage, statistical interpretation, and the scope of applicability. Most points are raised as requests for clarification or additional context rather than as fundamental objections. The reviewer indicates that the manuscript could be of interest to a broader audience if these application-related aspects are sufficiently clarified.

Assessment of the responses to Reviewer #1's comments:
Comment 1: The authors have improved the description of the dataset and clarified how the data were obtained and processed. However, the extent to which the specific measurements quantitatively support the analyses is only partially addressed, as no additional exploratory or sanity-check analyses of data variability are included.

Response:
As part of the Methods section, we have included a full description of each of the datasets considered, including number of replicates, variation in length of each organism and a description of the characteristics of the data. In the revised manuscript, we added a more detailed discussion to explain why each dataset was chosen, appearing in the Introduction for Drosophila embryos, Results section F for the gastruloids and neural tube data, and in Methods sections A-C. Beyond these textual descriptions, we have also added to the Methods statistics regarding the variation of the expression of key genes along the patterning axes for all systems considered. These show that the variation of the gene expression due to the different replicates is small relative to the magnitude of expression, with typical coefficients of variation (CV) on the scale of ~0.15-0.4. We also explored the CV of gene expression at peaks of pair-rule genes, which correspond with stripe positions in Drosophila embryos, and show that their CV also fall within the range of ~0.3-0.4. Furthermore, as part of Methods section A, we summarized findings from previous work which illustrate that the mutant background embryos have similar gene expression patterns as WT embryos, lending credibility towards our statistical analysis in Results section E. Finally, we have further strengthened the discussion regarding the limitations of the approach in the Discussion section, which further describe the applicability of our methodology on the datasets described in the revised text.

Comment 2: The statistical testing and the relationship between the reported p-values and the figures are clarified. The response focuses mainly on technical details of the tests and does not fully address the reviewer's point that these comparisons represent the primary ablation distinguishing this work from prior models.

Response:
To better distinguish this work from previous works, a large portion of the Introduction and of Results section A are now dedicated towards explaining the conceptual difference between our methodology and those previously explored. We explore the quantitative and qualitative implications of these differences in the Results section, and have further revised the text to more clearly describe in which ways our theoretical framework expands on previous models. In this vein, we have added text in the Introduction, Results section B, and the Discussion.

Comment 3: The concern regarding generality is addressed by extending the analysis to two additional developmental systems, providing independent support that the reported effects are not limited to a single dataset or organism.

Comment 4: The authors acknowledge the potential ambiguity between neighborhood effects and latent intrinsic determinants and limit the scope of their claims accordingly. Some abstract-level phrasing may still be interpreted as implying a causal relationship, depending on wording.

Response: Part of the Discussion section is now dedicated towards communicating the scope of our approach, and in particular we touch on the question of causal relationships where we state that our results are independent of the underlying mechanism, and do not imply a causal relationship related to the potential mechanism which relays information between neighboring cells. We have further revised key passages in the text to clarify this point and avoid ambiguity regarding the scope of our study.

Comment 5: The conceptual connection to graph-based spatial models is acknowledged and briefly discussed. Given the exploratory nature of the comment, this response appears sufficient.

Comment 6: The definitions of prediction and reconstruction errors are clarified in the main text and Methods, resolving the ambiguity raised by the reviewer.

Overall, the authors have responded constructively to the reviewer's comments and have addressed most points at an appropriate level. Several concerns are fully resolved through clarification of terminology, statistical testing, and the addition of analyses in additional biological systems. A small number of points remain only partially addressed and relate mainly to framing and interpretation rather than to missing analyses.
To further strengthen the revision without requesting new data, it may be helpful to ask the authors to (i) briefly note the limitations of the current measurements when motivating the quantitative analyses, (ii) state more explicitly in the main text that the comparison between neighborhood-informed and cell-independent decoders represents the main ablation underlying the framework, and (iii) review abstract-level wording to avoid or clearly qualify causal interpretations of neighborhood effects. These points could likely be addressed through targeted textual revisions in the Abstract, Results, and Discussion.

Response: We thank the reviewer for taking the time and effort to go through Reviewer 1's comments and our responses, and for explicitly directing our efforts in revising the manuscript. As suggested by the reviewer, we have revised the manuscript to:
(i) Better describe the limitations of the approach, as well as of current measurements, in the Introduction and Discussion sections, paired with the addition of a brief summary of the information theoretic approach towards positional information in the Supplementary Material.
(ii) More clearly contextualize the comparison to cell-independent decoders, in the Introduction and Results sections.

(iii) Reworked passages to clarify the extent of our work, and avoid text which could be misinterpreted as related to implications for specific cellular mechanisms, in the Abstract, Introduction and Discussion sections.

Reviewer 2 comments (partially quoted and partially paraphrased) on comments from Reviewer 3:

Please address Reviewer 3's conceptual concerns explicitly through targeted revision (rather than by introducing additional analyses), with the following points as guidance:

Response: We thank the reviewer for offering specific guidance on how to revise the manuscript in light of the new comments by reviewer 3.

(1) Conceptual framing and terminology. It would be helpful to revisit language that could be read as implying direct cell-cell interaction or molecular readout, and to state more explicitly that "neighborhood information" is meant to refer to statistical correlations within an information-theoretic decoding framework. The authors could also identify places where the statistical availability of information might be interpreted as biological accessibility at the single-cell level, and to clarify or temper these statements accordingly.

Response: We have revised and clarified core passages in the Abstract, Introduction and Discussion sections which could be misinterpreted as implying that our results imply a specific signaling mechanism or direct cell-cell interaction for the decoding of positional information. We believe that the revised text more accurately reflects the scope of our work in light of these comments.

(2) Methodological scope and interpretation. Please provide a clearer justification of the probabilistic decoding approach, including a brief discussion of why this framework is appropriate for the biological question being addressed, as well as an explicit acknowledgment of its limitations. It would also be useful for the authors to more clearly distinguish which conclusions are directly supported by the computational analysis and which should be presented as hypothesis-generating rather than mechanistically established.

Response: A large part of Results section A is dedicated to summarizing the reasoning behind the probabilistic approach taken in previous works and how our framework incorporates this probabilistic view. In addition to this, we added a section in the revised Supplementary Information (the new section A) to give a more in-depth summary of the methodology, reasoning, and common practices used in the framework of positional information. As mentioned, this new section includes an introduction to core concepts from the information-theoretic approach for estimating positional information, including a better contextualization of the necessity for probabilistic decoding within this framework. Furthermore, we have revised targeted passages in the Discussion section to more explicitly express our conceptual and computational contributions, in contrast to previous cell-independent

approaches, as well as added text regarding potential future work towards the interrogation of specific biological mechanisms in which the concepts of neighborhood-informed positional information might be carried out.

(3) Contextualization of prior work. The manuscript would benefit from a concise but explicit summary of the core findings of Dubuis et al. (2013) and McGough et al. (2024), so that it can be read as a more self-contained contribution. In addition, please clarify more precisely how their framework extends, reinterprets, or differs from these prior studies.

Response: As above, we have added a section to the Supplementary Information explicitly dedicated to summarizing core findings of previous work in the field, in particular that of Dubius et al. (2013) (SI section A1) and McGough (SI section A2). This new section provides a better contextualization of our framework, which is described in full detail in Results section A and in the Methods section.

(4) Biological scope and narrative limits. It may be important for the authors to clearly specify the developmental stages, spatial domains, and axes to which their analysis applies, and to avoid extrapolating beyond those boundaries. With regard to the mouse data, the authors could either strengthen the conceptual rationale for its inclusion or, alternatively, present it more explicitly as a limited illustrative extension rather than a central validation of the framework.

Response: Based on feedback from the previous revision round, we have elaborated on the limitations of the approach in the Introduction and Discussion sections. Beyond these notes, we also described the requirements needed to apply our approach, such as a clear, single axis of patterning, multiple replicates and high-quality measurements. Following this comment, we added additional text meant to more clearly specify where our approach can be used and how to interpret the outputs, as well as why we chose the particular systems in our work. In the revised text, we better describe the limitations related to working with the mouse data in the beginning of Results section F, and put our results in better context in the Discussion section, including limitations of the approach. Furthermore, the Introduction section establishes the advantage of considering the AP axis along Drosophila embryos, while new text in Results section F motivates our choices for the other datasets.

(5) Terminology and presentation. It would be beneficial to revise potentially confusing or overloaded terminology (for example, the use of “gap” in multiple contexts), which would help reduce conceptual ambiguity and improve clarity for a broad readership.

Response: The terminology for concepts such as “gap genes” and the “information gap” are well established in existing literature [1-4]. As such, we believe that exchanging these for other terms might cause even further confusion. Nonetheless, we agree that this jargon is overloaded and as such have taken steps to ensure that it is contextually clear which meaning is referenced whenever an overloaded term is used in the revised manuscript.

References:

1. Dubuis, Julien O., Reba Samanta, and Thomas Gregor. "Accurate measurements of dynamics and reproducibility in small genetic networks." Molecular systems biology 9.1 (2013): 639.
2. Dubuis, Julien O., et al. "Positional information, in bits." Proceedings of the National Academy of Sciences 110.41 (2013): 16301-16308.
3. McGough, Lauren, et al. "Finding the last bits of positional information." PRX life 2.1 (2024): 013016.
4. Petkova, Mariela D., et al. "Optimal decoding of cellular identities in a genetic network." Cell 176.4 (2019): 844-855.

26th Feb 2026

Manuscript Number: MSB-2025-12910RR
Title: Neighborhood-Informed Positional Information for Precise Cell Identity Specification
Author: Michal Erez
Roy Friedman
Mor Nitzan

Dear Dr Nitzan,

Thank you for the submission of your revised manuscript to Molecular Systems Biology. We have now received the enclosed report from Reviewer 2, who is supportive of publishing your manuscript at MSB, and I am pleased to inform you that we will be able to accept your manuscript pending the following final amendments:

1) In the main manuscript file, list the authors together and affiliations below the whole list with labels for each affiliation.
2) Please label the Abstract.
3) Please include keywords to max. 5.
4) Please combine the 'Code Availability' section into the 'Data availability' and ensure that it is formatted according to the example below. The Data availability statement should only include newly generated data and code; for re-use of publicly available data, we encourage Data citations (see below).
"The datasets and computer code produced in this study are available in the following databases:
- Chip-Seq data: Gene Expression Omnibus GSE46748 (https://www.ncbi.nlm.nih.gov/geo/query/acc.cgi?acc=GSE46748)
- Modeling computer scripts: GitHub (https://github.com/SysBioChalmers/GECKO/releases/tag/v1.0)
- [data type]: [full name of the resource] [accession number/identifier] ([doi or URL or identifiers.org/DATABASE:ACCESSION])"
5) Our journal encourages inclusion of *data citations in the reference list* to directly cite datasets that were re-used and obtained from public databases. Data citations in the article text are distinct from normal bibliographical citations and should directly link to the database records from which the data can be accessed. In the main text, data citations are formatted as follows: "Data ref: Smith et al, 2001" or "Data ref: NCBI Sequence Read Archive PRJNA342805, 2017". In the Reference list, data citations must be labeled with "[DATASET]". A data reference must provide the database name, accession number/identifiers and a resolvable link to the landing page from which the data can be accessed at the end of the reference. Further instructions are available at .
6) Please rename "Competing Interests" to "Disclosure and competing interests statement". We updated our journal's competing interests policy in January 2022 and request authors to consider both actual and perceived competing interests. Please review the policy https://link.springer.com/partners/embo-press/editorial-policies#Competing%20interest%20disclosures and update your competing interests if necessary.
7) Please correct the reference citation in the reference list to be alphabetical (not numerical). Where there are more than 10 authors on a paper, only the first 10 should be listed, followed by "et al.". Please check "Author Guidelines" for more information: https://link.springer.com/journal/44320/submission-guidelines#cms-Reference-guidelines
8) Please place individual sections of the manuscript in the following order: Title page - Abstract & Keywords - Introduction - Results - Discussion - Methods - Data Availability - Acknowledgements - Disclosure and Competing Interests Statement - References - Figure Legends - Expanded View Figure Legends.
9) For the figures and figure legends, please take care of the following:
- Please remove all figures from main manuscript file and leave only main figure legends placed after the references.
- Please make sure to update the callouts of all figures in the main manuscript text such that they appear sequentially. Currently figure callouts are missing for Figure 4c.
- For Figure S3 the panels a-f need to be included in the figure caption.
- Please note that the exact p values need to be provided in the legends or figures of figures 2D, E; 3D, 4E, S2, S4 B, C
- Please note that the box plots need to be defined in terms of minima, maxima, centre, bounds of box and whiskers, and percentile in the legends of figures 3C, 4D, S2
- Please note that information related to n is missing in the legends of figures 2D, E; 3C, D, 4D, 5C, F; S4 B, C
- Please note that the error bars are not defined in the legends of figures 2D, 5F, S4 B
- Please note that the measure of center for the error bars needs to be defined in the legends of figures 2E, 4E, S4 C
10) Appendix file: Please remove the "Supplementary Information" from the main manuscript file and upload it as an Appendix as a single PDF (no separate image files are needed for Appendix figures). Please include a title page with the label "Appendix for + manuscript title" and a Table of Contents with page numbers for the listed items. The nomenclature should be Appendix Figure Sx and Appendix Table Sx throughout manuscript and Appendix PDF, and please be sure to update the callouts for the Supplementary Information and individual figures in the main manuscript file.
11) Please reorganize the synopsis figure so that it may fit into our required dimensions of 550 pixels wide x (300-600) pixels high (currently it is too wide).
12) Please provide the Source Data in formats that can be opened by common softwares (e.g. preferably .xls).
13) As part of the EMBO Publications transparent editorial process initiative (see our policy here:

https://www.embopress.org/transparent-process#Review_Process), Molecular Systems Biology will publish online a Peer Review File (PRF) to accompany accepted manuscripts. This file will be published in conjunction with your paper and will include the anonymous referee reports, your point-by-point response and all pertinent correspondence relating to the manuscript. Let us know whether you agree with the publication of the PRF and as here, if you want to remove or not any figures from it prior to publication. Please note that the Authors checklist will be published at the end of the PRF.
14) After your paper is published, we may promote it on social media. If you have any handles or hashtags for Bluesky you would like included, please let us know.
15) Please provide a point-by-point letter INCLUDING my comments as well as the reviewer's final comments and your detailed responses (as Word file).

I look forward to reading a new revised version of your manuscript as soon as possible.

Yours sincerely,

Poonam Bheda, PhD
Scientific Editor
Molecular Systems Biology

Reviewer #2:

Overall, I think the authors have responded well to the main points I raised from Reviewer 3's comments, and the manuscript is improved accordingly.

In particular, they have toned down wording that could be taken to imply a specific biological mechanism or direct cell-cell interactions, and they explain more explicitly what they mean by "neighborhood information" in an information-theoretic decoding sense. They also explain the probabilistic decoding approach more clearly, and they are more careful about distinguishing what the analysis directly supports from what is more speculative. Finally, the added summaries of prior work and the clearer statements of scope across datasets provide a clearer context for how the work relates to prior studies and where its conclusions apply.

I just have one wording suggestion: "with 1% accuracy" could be misleading. The cited result is better described as ~1% embryo-length positional precision (or positional uncertainty/error) along the A-P axis.

Dear Dr. Poonam Bheda,

We again thank all reviewers for their constructive comments and positive evaluation of our manuscript. We have addressed all remaining comments in the point-by-point response below, and have revised the manuscript accordingly.

Sincerely,
Michal, Roy, and Mor

Editor's items:

1) In the main manuscript file, list the authors together and affiliations below the whole list with labels for each affiliation.
Response: Done.

2) Please label the Abstract.
Response: Done.

3) Please include keywords to max. 5.
Response: Done.

4) Please combine the 'Code Availability' section into the 'Data availability' and ensure that it is formatted according to the example below. The Data availability statement should only include newly generated data and code; for re-use of publicly available data, we encourage Data citations (see below).
"The datasets and computer code produced in this study are available in the following databases:
- Chip-Seq data: Gene Expression Omnibus GSE46748 (https://www.ncbi.nlm.nih.gov/geo/query/acc.cgi?acc=GSE46748)
- Modeling computer scripts: GitHub (https://github.com/SysBioChalmers/GECKO/releases/tag/v1.0)
- [data type]: [full name of the resource] [accession number/identifier] ([doi or URL or identifiers.org/DATABASE:ACCESSION])"

Response: Done.

5) Please rename "Competing Interests" to "Disclosure and competing interests statement". We updated our journal's competing interests policy in January 2022 and request authors to consider both actual and perceived competing interests. Please review the policy https://link.springer.com/partners/embo-press/editorial-policies#Competing%20interest%20disclosures and update your competing interests if necessary.
Response: Done.

6) Please correct the reference citation in the reference list to be alphabetical (not numerical). Where there are more than 10 authors on a paper, only the first 10 should be listed, followed by "et al.". Please check "Author Guidelines" for more information:
https://link.springer.com/journal/44320/submission-guidelines#cms-Reference-guidelines
Response: Done.

7) Please place individual sections of the manuscript in the following order: Title page - Abstract & Keywords - Introduction - Results - Discussion - Methods - Data Availability - Acknowledgements - Disclosure and Competing Interests Statement - References - Figure Legends - Expanded View Figure Legends.
Response: Done.

8) For the figures and figure legends, please take care of the following:
- Please remove all figures from main manuscript file and leave only main figure legends placed after the references.
- Please make sure to update the callouts of all figures in the main manuscript text such that they appear sequentially. Currently figure callouts are missing for Figure 4c.
- For Figure S3 the panels a-f need to be included in the figure caption.
- Please note that the exact p values need to be provided in the legends or figures of figures 2D, E; 3D, 4E, S2, S4 B, C
- Please note that the box plots need to be defined in terms of minima, maxima, centre, bounds of box and whiskers, and percentile in the legends of figures 3C, 4D, S2
- Please note that information related to n is missing in the legends of figures 2D, E; 3C, D, 4D, 5C, F; S4 B, C
- Please note that the error bars are not defined in the legends of figures 2D, 5F, S4 B
- Please note that the measure of center for the error bars needs to be defined in the legends of figures 2E, 4E, S4 C
Response: Done.

9) Appendix file: Please remove the "Supplementary Information" from the main manuscript file and upload it as an Appendix as a single PDF (no separate image files are needed for Appendix figures). Please include a title page with the label "Appendix for + manuscript title" and a Table of Contents with page numbers for the listed items. The nomenclature should be Appendix Figure Sx and Appendix Table Sx throughout manuscript and Appendix PDF, and please be sure to update the callouts for the Supplementary Information and individual figures in the main manuscript file.
Response: Done.

10) Please reorganize the synopsis figure so that it may fit into our required dimensions of 550 pixels wide x (300-600) pixels high (currently it is too wide).
Response: Done.

11) Please provide the Source Data in formats that can be opened by common softwares (e.g. preferably .xls).

Response: Done.

Reviewer 2 comments:

Overall, I think the authors have responded well to the main points I raised from Reviewer 3's comments, and the manuscript is improved accordingly.

In particular, they have toned down wording that could be taken to imply a specific biological mechanism or direct cell-cell interactions, and they explain more explicitly what they mean by "neighborhood information" in an information-theoretic decoding sense. They also explain the probabilistic decoding approach more clearly, and they are more careful about distinguishing what the analysis directly supports from what is more speculative. Finally, the added summaries of prior work and the clearer statements of scope across datasets provide a clearer context for how the work relates to prior studies and where its conclusions apply.

Response: We thank the reviewer for the constructive suggestions over the course of the review process, and we are glad to hear that the new manuscript is improved and more clearly describes our contributions.

I just have one wording suggestion: "with 1% accuracy" could be misleading. The cited result is better described as ~1% embryo-length positional precision (or positional uncertainty/error) along the A-P axis.
Response: We have adjusted the wording of the relevant passage of the Abstract, following the reviewer's suggestion.

15th Apr 2026
Manuscript number: MSB-2025-12910RRR
Title: Neighborhood-Informed Positional Information for Precise Cell Identity Specification

Dear Dr Nitzan,

Thank you again for sending us your revised manuscript. We are now satisfied with the modifications made and I am pleased to inform you that your paper has been accepted for publication.

Your manuscript will be processed for publication by EMBO Press. It will be copy edited and you will receive page proofs prior to publication. Please note that you will be contacted by Springer Nature Author Services to complete licensing and payment information.

You may qualify for financial assistance for your publication charges - either via a Springer Nature fully open access agreement or an EMBO initiative. Check your eligibility: https://link.springer.com/journal/44320/how-to-publish-with-us

Should you be planning a Press Release on your article, please get in contact with embo_production@springernature.com as early as possible in order to coordinate publication and release dates.

If you have any questions, please do not hesitate to contact the Editorial Office. Thank you for your contribution to Molecular Systems Biology.

Yours sincerely,

Sincerely,

Poonam Bheda, PhD
Scientific Editor
Molecular Systems Biology

>>> Please note that it is Molecular Systems Biology policy for the transcript of the editorial process (containing referee reports and your response letter) to be published as an online supplement to each paper. If you do NOT want this, you will need to inform the Editorial Office via email immediately. More information is available here: https://link.springer.com/partners/embo-press/editorial-policies#Peer%20review

EMBO Press Author Checklist

Corresponding Author Name: **Mor Nitzan**
Journal Submitted to: **Molecular Systems Biology**
Manuscript Number: **MSB-2025-12910**

USEFUL LINKS FOR COMPLETING THIS FORM
The EMBO Journal - Author Guidelines
EMBO Reports - Author Guidelines
Molecular Systems Biology - Author Guidelines
EMBO Molecular Medicine - Author Guidelines

Reporting Checklist for Life Science Articles (updated January
This checklist is adapted from Materials Design Analysis Reporting (MDAR) Checklist for Authors. MDAR establishes a minimum set of requirements in transparent reporting in the life sciences (see Statement of Task: 10.31222/osf.io/9sm4x). Please follow the journal's guidelines in preparing your manuscript.
Please note that a copy of this checklist will be published alongside your article.

Abridged guidelines for figures
1. Data
The data shown in figures should satisfy the following conditions:
- the data were obtained and processed according to the field's best practice and are presented to reflect the results of the experiments in an accurate and unbiased manner.
- ideally, figure panels should include only measurements that are directly comparable to each other and obtained with the same assay.
- plots include clearly labeled error bars for independent experiments and sample sizes. Unless justified, error bars should not be shown for technical
- if n<5, the individual data points from each experiment should be plotted. Any statistical test employed should be justified.
- Source Data should be included to report the data underlying figures according to the guidelines set out in the authorship guidelines on Data

2. Captions
Each figure caption should contain the following information, for each panel where they are relevant:
- a specification of the experimental system investigated (eg cell line, species name).
- the assay(s) and method(s) used to carry out the reported observations and measurements.
- an explicit mention of the biological and chemical entity(ies) that are being measured.
- an explicit mention of the biological and chemical entity(ies) that are altered/varied/perturbed in a controlled manner.
- the exact sample size (n) for each experimental group/condition, given as a number, not a range;
- a description of the sample collection allowing the reader to understand whether the samples represent technical or biological replicates (including how many animals, litters, cultures, etc.).
- a statement of how many times the experiment shown was independently replicated in the laboratory.
- definitions of statistical methods and measures:

  - common tests, such as t-test (please specify whether paired vs. unpaired), simple χ2 tests, Wilcoxon and Mann-Whitney tests, can be unambiguously identified by name only, but more complex techniques should be described in the methods section;

  - are tests one-sided or two-sided?

  - are there adjustments for multiple comparisons?
  - exact statistical test results, e.g., P values = x but not P values < x;
  - definition of 'center values' as median or average;
  - definition of error bars as s.d. or s.e.m.

Please complete ALL of the questions below.
Select "Not Applicable" only when the requested information is not relevant for your study.

Materials

Newly Created Materials	Information included in the manuscript?	In which section is the information available? (Reagents and Tools Table, Materials and Methods, Figures, Data Availability Section)
New materials and reagents need to be available; do any restrictions apply?	Not Applicable	

Antibodies	Information included in the manuscript?	In which section is the information available? (Reagents and Tools Table, Materials and Methods, Figures, Data Availability Section)
For **antibodies** provide the following information:<br>- Commercial antibodies: RRID (if possible) or supplier name, catalogue number and or/clone number<br>- Non-commercial: RRID or citation	Not Applicable	

DNA and RNA sequences	Information included in the manuscript?	In which section is the information available? (Reagents and Tools Table, Materials and Methods, Figures, Data Availability Section)
**Short novel DNA or RNA including primers, probes**: provide the sequences.	Not Applicable	

Cell materials	Information included in the manuscript?	In which section is the information available? (Reagents and Tools Table, Materials and Methods, Figures, Data Availability Section)
**Cell lines:** Provide species information, strain. Provide accession number in repository **OR** supplier name, catalog number, clone number, and/**OR** RRID.	Not Applicable	
**Primary cultures:** Provide species, strain, sex of origin, genetic modification status.	Not Applicable	
Report if the cell lines were recently **authenticated** (e.g., by STR profiling) and tested for mycoplasma contamination.	Not Applicable	

Experimental animals	Information included in the manuscript?	In which section is the information available? (Reagents and Tools Table, Materials and Methods, Figures, Data Availability Section)
**Laboratory animals or Model organisms:** Provide species, strain, sex, age, genetic modification status. Provide accession number in repository **OR** supplier name, catalog number, clone number, **OR** RRID.	Not Applicable	
**Animal observed in or captured from the field:** Provide species, sex, and age where possible.	Not Applicable	
Please detail **housing and husbandry conditions**.	Not Applicable	

Plants and microbes	Information included in the manuscript?	In which section is the information available? (Reagents and Tools Table, Materials and Methods, Figures, Data Availability Section)
**Plants:** provide species and strain, ecotype and cultivar where relevant, unique accession number if available, and source (including location for collected wild specimens).	Not Applicable	
**Microbes:** provide species and strain, unique accession number if available, and source.	Not Applicable	

Human research participants	Information included in the manuscript?	In which section is the information available? (Reagents and Tools Table, Materials and Methods, Figures, Data Availability Section)
If collected and within the bounds of privacy constraints report on age, sex and gender or ethnicity for all study participants.	Not Applicable	

Core facilities	Information included in the manuscript?	In which section is the information available? (Reagents and Tools Table, Materials and Methods, Figures, Data Availability Section)
If your work benefited from core facilities, was their service mentioned in the acknowledgments section?	Not Applicable	

Design

Study protocol	Information included in the manuscript?	In which section is the information available? (Reagents and Tools Table, Materials and Methods, Figures, Data Availability Section)
If study protocol has been **pre-registered, provide DOI in the manuscript**. For clinical trials, provide the trial registration number **OR** cite DOI.	Not Applicable	

Report the **clinical trial registration number** (at ClinicalTrials.gov or equivalent), where applicable.	Not Applicable	

**Laboratory protocol**	**Information included in the manuscript?**	**In which section is the information available?** (Reagents and Tools Table, Materials and Methods, Figures, Data Availability Section)
Provide DOI OR other citation details if **external detailed step-by-step protocols** are available.	Not Applicable	

**Experimental study design and statistics**	**Information included in the manuscript?**	**In which section is the information available?** (Reagents and Tools Table, Materials and Methods, Figures, Data Availability Section)
Include a statement about **sample size** estimate even if no statistical methods were used.	Yes	Materials and Methods
Were any steps taken to minimize the effects of subjective bias when allocating animals/samples to treatment (e.g. **randomization procedure**)? If yes, have they been described?	Not Applicable	
Include a statement about **blinding** even if no blinding was done.	Not Applicable	
Describe **inclusion/exclusion criteria** if samples or animals were excluded from the analysis. Were the criteria pre-established?<br><br>If sample or data points were omitted from analysis, report if this was due to attrition or intentional exclusion and provide justification.	Not Applicable	
For every figure, are **statistical tests** justified as appropriate? Do the data meet the assumptions of the tests (e.g., normal distribution)? Describe any methods used to assess it. Is there an estimate of variation within each group of data? Is the variance similar between the groups that are being statistically compared?	Yes	Figures and associated captions, Materials and Methods

**Sample definition and in-laboratory replication**	**Information included in the manuscript?**	**In which section is the information available?** (Reagents and Tools Table, Materials and Methods, Figures, Data Availability Section)
In the figure legends: state number of times the experiment was **replicated** in laboratory.	Not Applicable	
In the figure legends: define whether data describe **technical or biological replicates**.	Not Applicable	

Ethics

**Ethics**	**Information included in the manuscript?**	**In which section is the information available?** (Reagents and Tools Table, Materials and Methods, Figures, Data Availability Section)
Studies involving **human participants**: State details of **authority granting ethics approval** (IRB or equivalent committee(s), provide reference number for approval.	Not Applicable	
Studies involving **human participants**: Include a statement confirming that **informed consent** was obtained from all subjects and that the experiments conformed to the principles set out in the WMA Declaration of Helsinki and the Department of Health and Human Services Belmont Report.	Not Applicable	
Studies involving **human participants:** For publication of **patient photos**, include a statement confirming that consent to publish was obtained.	Not Applicable	
Studies involving experimental **animals**: State details of **authority granting ethics approval** (IRB or equivalent committee(s), provide reference number for approval. Include a statement of compliance with ethical regulations.	Not Applicable	
Studies involving **specimen and field samples:** State if relevant **permits** obtained, provide details of authority approving study; if none were required, explain why.	Not Applicable	

**Dual Use Research of Concern (DURC)**	**Information included in the manuscript?**	**In which section is the information available?** (Reagents and Tools Table, Materials and Methods, Figures, Data Availability Section)
Could your study fall under dual use research restrictions? Please check biosecurity documents and list of **select agents and toxins** (CDC): https://www.selectagents.gov/sat/list.htm	Not Applicable	
If you used a select agent, is the security level of the lab appropriate and reported in the manuscript?	Not Applicable	
If a study is subject to dual use research of concern regulations, is the name of the **authority granting approval and reference number** for the regulatory approval provided in the manuscript?	Not Applicable	

Reporting

The MDAR framework recommends adoption of discipline-specific guidelines, established and endorsed through community initiatives. Journals have their own policy about requiring specific guidelines and recommendations to complement MDAR.

**Adherence to community standards**	**Information included in the manuscript?**	**In which section is the information available?** (Reagents and Tools Table, Materials and Methods, Figures, Data Availability Section)
State if relevant guidelines or checklists (e.g., **ICMJE, MIBBI, ARRIVE, PRISMA**) have been followed or provided.	Not Applicable	
For **tumor marker prognostic studies,** we recommend that you follow the **REMARK** reporting guidelines (see link list at top right). See author guidelines, under 'Reporting Guidelines'. Please confirm you have followed these guidelines.	Not Applicable	
For **phase II and III randomized controlled trials**, please refer to the **CONSORT** flow diagram (see link list at top right) and submit the CONSORT checklist (see link list at top right) with your submission. See author guidelines, under 'Reporting Guidelines'. Please confirm you have submitted this list.	Not Applicable	

Data Availability

**Data availability**	**Information included in the manuscript?**	**In which section is the information available?** (Reagents and Tools Table, Materials and Methods, Figures, Data Availability Section)
Have **primary datasets** been deposited according to the journal's guidelines (see 'Data Deposition' section) and the respective accession numbers provided in the Data Availability Section?	Not Applicable	
Were **human clinical and genomic datasets** deposited in a public access-controlled repository in accordance to ethical obligations to the patients and to the applicable consent agreement?	Not Applicable	
Are **computational models** that are central and integral to a study available without restrictions in a machine-readable form? Were the relevant accession numbers or links provided?	Yes	Code Availability Section
If publicly available data were reused, provide the respective **data citations in the reference list**.	Yes	Materials and Methods and Main Text
